# Effect of behavioural sleep interventions on blood pressure, heart rate, and heart rate variability in adults with poor sleep health: a systematic review, meta-analysis, and meta-regression analysis

**DOI:** 10.1093/ehjopen/oeag006

**Published:** 2026-01-20

**Authors:** Samiul A Mostafa, Wasim Hanif, George Balanos, Krishnarajah Nirantharakumar, Jason G Ellis, Abd A Tahrani

**Affiliations:** Department of Metabolism and Systems Science, IBR Tower, Level 2, College of Medicine and Health, University of Birmingham, Edgbaston, Birmingham B15 2TT, UK; Department of Diabetes, Nuffield House, Queen Elizabeth Hospital Birmingham, University Hospitals Birmingham NHS Foundation Trust, Mindelsohn Way, Edgbaston, Birmingham B15 2WB, UK; Centre of Endocrinology, Diabetes and Metabolism (CEDAM), Birmingham Health Partner, Nuffield House, Queen Elizabeth Hospital Birmingham, Mindelsohn Way, Edgbaston, Birmingham B15 2WB, UK; Department of Metabolism and Systems Science, IBR Tower, Level 2, College of Medicine and Health, University of Birmingham, Edgbaston, Birmingham B15 2TT, UK; Department of Diabetes, Nuffield House, Queen Elizabeth Hospital Birmingham, University Hospitals Birmingham NHS Foundation Trust, Mindelsohn Way, Edgbaston, Birmingham B15 2WB, UK; Centre of Endocrinology, Diabetes and Metabolism (CEDAM), Birmingham Health Partner, Nuffield House, Queen Elizabeth Hospital Birmingham, Mindelsohn Way, Edgbaston, Birmingham B15 2WB, UK; School of Sport, Exercise and Rehabilitation Sciences (SportEx), University of Birmingham, Edgbaston campus, Birmingham B15 2TT, UK; Centre of Endocrinology, Diabetes and Metabolism (CEDAM), Birmingham Health Partner, Nuffield House, Queen Elizabeth Hospital Birmingham, Mindelsohn Way, Edgbaston, Birmingham B15 2WB, UK; Institute of Applied Health Research, Public Health Building, University of Birmingham, Edgbaston, Birmingham B15 2TT, UK; Midlands Health Data Research UK, 2nd Floor, Mindelsohn Way, Edgbaston, Birmingham B15 2TH, UK; Department of Psychology, Northumberland Building, City Campus, University of Northumbria, Newcastle upon Tyne NE1 8ST, UK; Department of Metabolism and Systems Science, IBR Tower, Level 2, College of Medicine and Health, University of Birmingham, Edgbaston, Birmingham B15 2TT, UK; Department of Diabetes, Nuffield House, Queen Elizabeth Hospital Birmingham, University Hospitals Birmingham NHS Foundation Trust, Mindelsohn Way, Edgbaston, Birmingham B15 2WB, UK; Centre of Endocrinology, Diabetes and Metabolism (CEDAM), Birmingham Health Partner, Nuffield House, Queen Elizabeth Hospital Birmingham, Mindelsohn Way, Edgbaston, Birmingham B15 2WB, UK

**Keywords:** Poor sleep, Sleep disorder, Intervention, Blood pressure, Heart rate, Meta-analysis

## Abstract

**Background:**

Poor sleep health is known to negatively impact cardiovascular risk factors, including systolic (SBP) and diastolic blood pressure (DBP), heart rate (HR), and heart rate variability (HRV). Consequently, there is interest in determining the impact of improving sleep on cardiovascular risk. We reviewed studies aimed at improving sleep using (1) Cognitive Behavioural Therapy for Insomnia (CBT-I) and/or sleep hygiene and (2) sleep extension on these risk factors.

**Methods:**

Literature searches were performed on MEDLINE, EMBASE, CINAHL, and Cochrane Library. Studies featuring adults ≥ 18years, a sleep intervention and pre and postrisk factor measurements available were included. Studies of obstructive sleep apnoea were excluded.

**Results:**

From 21 studies (*n* = 1222), meta-analyses of 12 RCTs (*n* = 688), demonstrated a significant reduction in SBP averaging 4.91 mmHg [2.38, 7.43, *P* < 0.00001, heterogeneity (I^2^) = 74%], compared to control. When 15 RCTs and non-RCTs were combined (*n* = 860), reductions in SBP and DBP averaged 5.02 mmHg (95% CI 2.80, 7.23, *P* < 0.00001, *I*^2^ 67%) and 2.90 mmHg (0.30, 5.49; *P* = 0.03, *I*^2^ 88%), respectively. In eight CBT-I and/or sleep hygiene interventions (*n* = 618), the SBP decrease averaged 3.44 mmHg (1.07, 5.80, *P* = 0.004). In sleep extension interventions (*n* = 242; 7 studies), reductions in SBP averaged 7.59 mmHg (4.74, 10.44; *P* < 0.00001), DBP 4.83 mmHg (0.73, 8.92; *P* = 0.02), and HR (*n* = 164, 4 studies) 1.24 beats/minute (0.44, 2.44; *P* = 0.04). No significant changes in HRV were observed. Seven studies were of low concern in the quality assessment.

**Conclusions:**

Using behavioural sleep interventions led to clinically significant reductions in blood pressure, suggesting addressing poor sleep health could feature in blood pressure management. Future randomized controlled trials are still required.

**PROSPERO Identification number:**

CRD42025628290

## Graphical Abstract

**Graphical Abstract:**
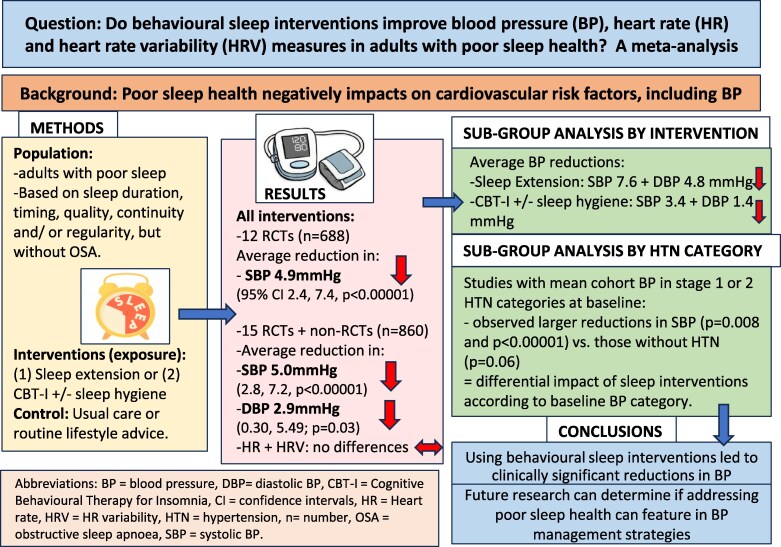

## Introduction

Cardiovascular disease (CVD) remains a public health concern, despite the introduction of multiple interventions to improve precursor risk factors, including hypertension. Accordingly, the emergence of a separate epidemic, poor or suboptimal sleep health, which affects over 30–40% of adults, also increases the risk of CVD.^1–7^ Such individuals exhibit subclinical dimensions of sleep health related to one or more likely a combination of sleep timing, quality, duration, continuity, and/or regularity that can occur within or without a diagnosed sleep disorder. The dimensions tend to overlap as they can trigger or exacerbate one another (e.g. co-morbid insomnia and OSA or short sleep duration and insomnia). Poor sleep health is independently associated with CVD risk factors, including hypertension, type 2 diabetes mellitus (T2DM), and, through autonomic dysregulation, adverse levels of heart rate and reduced heart rate variability.^3–8^ Consequently, there has been recent interest in examining whether addressing poor sleep health as a whole reduces the impact of these risk factors.

The principal options for treating poor sleep health include pharmacological therapies, continuous positive airway pressure (CPAP) and mandibular devices for Obstructive Sleep Apnoea (OSA), bright light therapy, and work schedule changes for rotating and night-shift workers.^9,10^ Furthermore, behavioural sleep interventions are known to be feasible and have some advantages as they do not involve taking medications or using breathing equipment. These are usually centred on (i) sleep extension, which aims to increase the length of sleep duration or time-in-bed, (ii) Cognitive Behaviour Therapy for Insomnia (CBT-I), which focuses on restructuring thoughts and behaviours that contribute to poor sleep health, and (iii) sleep hygiene/education/counselling, which provide advice on good sleep habits and routines. There is often some cross-over in the contents of these interventions, so they can address more than one subclinical sleep dimension. For example, modern CBT-I interventions contain a module on sleep hygiene advice. Also, as insomnia and short sleep duration can co-exist, sometimes sleep extension can be added to CBT-I.

These interventions are known to improve sleep health and demonstrate benefits on some CVD risk factors such as glycaemia.^11^ Less is known about the impact of these interventions on other CVD risk factors such as blood pressure and heart rate. As rates of hypertension remain uncontrolled in up to 80% of adults, despite the use of multiple antihypertensive therapies, there is a need to investigate novel interventions that could improve this and other CVD risk factors.^12^

Two previous meta-analyses did not demonstrate any significant impact of behavioural sleep interventions on systolic or diastolic blood pressure; however, few studies were available at the time and the quality of the included studies was largely poor.^13,14^ Moreover, heart rate and heart rate variability have not been investigated, to date, in meta-analyses. As more published studies have become available in this field, we wished to investigate this further. We hypothesized sleep interventions would have a differential impact on risk factors according to their baseline severity, with greater benefits observed at more adverse baseline levels (e.g. stage 1 or 2 hypertension compared to normal blood pressure), which would follow the results observed for glycaemia.^11^

The aim of this systematic review and meta-analysis was to assess the impact of behavioural sleep interventions on systolic and diastolic blood pressure, heart rate and heart rate variability.

## Methods

This systematic review was registered on International Prospective Registry of Systematic Reviews (PROSPERO Identification number: CRD42025628290) database and is compliant with the requirements of the Preferred Reporting Items for Systematic Reviews and Meta-Analyses statement.^15^

### Information sources and search strategy

The search strategy was developed by SM in collaboration with an experienced local hospital health sciences librarian to identify relevant studies using MEDLINE, Embase, CINAHL, and Cochrane Library. The electronic search strategy used for all databases is included in Supplementary material online, *Appendix A*. The literature search was performed from database inception up to 19th December 2024. Subsequently, using RefWorks reference management software, duplicates were removed, and then two investigators (SM and WH) independently screened and assessed titles and abstracts of identified studies for eligibility. The full text of these papers was retrieved and independently assessed for inclusion. Discrepancies were resolved through a third investigator (AT) where necessary, until a consensus was reached. The reference lists of review articles and of included original publications were also screened for potentially relevant studies. We focused on studies published as full-text papers, of any language, but we also considered abstract publications, if further information/data could be provided by the original authors and/or were published in subsequent meta-analyses.

### Study selection and eligibility criteria

We included intervention studies that were (a) randomized controlled trials (RCTs), non-randomized trials, quasi experimental, laboratory, or single-arm cross-over (‘pre–post’) intervention studies, (b) consisted of nonpregnant adults ≥18 years old at baseline, (c) with a sleep intervention as the exposure (CBT-I and/or sleep hygiene/education/counselling or sleep extension), and (d) measured both pre and postintervention systolic and diastolic blood pressure, heart rate, and/or heart rate variability markers. The preferred format of blood pressure and heart rate measurements was 24-h average results; however, we accepted single measurements if taken in a resting state. The blood pressure readings could be brachial or central aortic. The measurements could be made using various equipment, including a sphygmomanometer (measured manually or electronically) or digital monitors (e.g. specialized wrist watches).

For two-arm studies, the comparator was considered as either a control group (i.e. no intervention), a lifestyle programme, or an advice session consisting of diet and exercise modifications (considered as standard care). If the sleep intervention was part of a multifactorial lifestyle intervention, the study was included only if the control arm consisted of the same lifestyle intervention without the sleep component. For this reason, in the case of any three-arm studies, we included data from two arms.

The exclusion criteria consisted of studies that (1) did not feature a sleep intervention, (2) included people with OSA, (3) consisted of sleep interventions for CPAP (or similar procedure) for OSA, bright/dim light exposure, drug therapies or laboratory-based sleep restriction/deprivation as the exposure; (4) did not report measures of the outcome at both baseline and follow-up; or (5) focused on pulmonary hypertension.

### Data extraction

One investigator (SM) extracted the data and the second investigator (WH) independently checked for consistency. Data was extracted on the following: first author’s surname, year of publication, country of origin of the population studied, type of study (e.g. randomized trial), sample size of intervention and control arms, population demographic characteristics (e.g. age, sex, body mass index), sleep variable(s) under analysis (subjective and/or objective measures), type of sleep intervention, length of follow-up, measure(s) used to assess change in sleep variable, type of outcome variable(s) measured, and changes in the levels of the sleep and outcome variables at baseline and follow-up.

Sleep interventions were placed into two categories. First, we used (a) CBT-I and/or sleep hygiene, which have a common focus on improving outcomes of sleep quality or insomnia symptoms (e.g. Pittsburgh Sleep Quality Index, PSQI global score, and Insomnia Severity Index, ISI). Also, sleep hygiene is routinely incorporated into modern CBT-I interventions. Secondly, (b) sleep extension, which focuses on increasing sleep duration (SLD), total sleep time (TST), and time in bed (TIB). These two categories follow those used in previous systematic reviews.^11,16^

### Study risk of bias assessment

Study quality assessment was performed on studies by two reviewers (SM and WH), according to the Cochrane risk-of-bias tool for randomized trials version 2 (ROB-2) for parallel arm or cross-over RCTs or using the Risk-of-bias in non-randomized studies of interventions (ROBINS-I) tool for single-arm crossover studies.^17,18^ Reviewers met to compare results and reach a consensus. ROB-2 includes five assessments of possible bias, including randomization process, deviation from proposed interventions, missing outcome data, outcome measurement, and result reporting. ROBINS-I has seven assessments of potential bias of confounding, selection, intervention classification, deviation of interventions, missing data, outcome measurement, and result reporting. Studies were judged to have low, some, or high risk/concern of bias for each assessment. The results of the risk of bias assessments were converted into traffic-light and weighted summary plots.

### Statistical analysis

Where sufficient information existed, we analysed data quantitatively using meta-analysis. Initially, we focused on RCTs and non-RCTs separately, before combining these together. The non-RCTs were ‘pre and post’-single arm cross-over studies where the same population of participants underwent the first phase with no intervention (considered control), then undertook the sleep intervention (considered intervention), followed by final measurements.

The pooled results from all sleep interventions were analysed firstly together and then separately in a subgroup analysis as studies of (1) sleep extension or (2) CBT-I and/or sleep hygiene. In cases where studies reported data as both intention-to-treat and per protocol data, we selected intention-to-treat to minimize the impact of bias.

Secondly, we analysed the pooled results according to the baseline blood pressure categories of individual studies of normal blood pressure, elevated, and stages 1 and 2 hypertension using the 2017 ACC/AHA guidelines.^19^ If precise information was not available on the percentage distribution of blood categories at baseline in each study, we proceeded to perform categorization of each study according to the mean cohort blood pressure levels at baseline.

The outcome variables were reported as mean (standard deviation, SD). When the original study reported outcomes as median (interquartile range, IQR) or mean (95% confidence intervals, 95% CI, or standard error), these were converted to mean (SD) using accepted methods.^20^ We estimated the pooled mean differences (95% CI) of the variables of interest, considered as the effect size, for outcomes of blood pressure and heart rate. For heart rate variability, we envisaged some studies may report the indices in the ‘non-normalized’ form, thus we used standardized mean differences instead. These indices relate to the time domain of the SD of normal-to-normal intervals (SDNN) and the frequency domains of high-frequency (HF) and the low frequency: high frequency (LF:HF) power ratio. Better heart rate variability was indicated with higher SDNN and HF levels and a lower LF:HF ratio, representing surrogate measures for higher parasympathetic and lower sympathetic activity.

Pooled differences were calculated using the inverse variance method and through the use of a random-effects model, as we anticipated a degree of study-level variability in the exact interventions used. Heterogeneity among studies was quantified by *I*^2^ statistic. We considered low, moderate, and high *I*^2^ values to be 25, 50, and 75%, respectively.^21^

Sensitivity analyses were performed to check for robustness of data for the main outcomes of the meta-analysis. First, we investigated the possible risk of publication bias of smaller studies on outcomes where a sufficient number of studies were present. Studies with less than 60 participants were removed and the main outcome results were re-analysed. This was complemented by visually inspecting funnel plots. Secondly, a ‘leave-one-out’ analysis was conducted for the meta-analysis results by removing one study at a time from the dataset and before re-running the meta-analysis to see how the overall results changed. Finally, to assess the overall impact of risk of bias on the main outcomes, studies within the meta-analyses were categorized according to allocated ranking (low risk, some concern, or high risk) before re-running the meta-analysis for categories that contained three or more studies.

#### Meta-regression analysis

Where the pooled prevalence of more than 10 studies via meta-analysis had moderate or high heterogeneity, meta-regression models were produced to identify any confounding variables that could have influenced the effect of the interventions on the outcome. A total of 13 univariate meta-regression analyses were conducted using a random effects model. Covariates considered were age, BMI, sex, geographical location (North America vs. other), study design (RCT vs. non-RCT), intervention type, year of publication, intervention duration, intervention delivery (remote vs. face-to-face), baseline systolic or diastolic blood pressure level, baseline blood pressure category based on the 2017 ACC/AHA guidelines^19^ and measurement method (single spot vs. average 24-h reading). Statistical analyses were performed with Review Manager (RevMan) software Version 5.4.1 (Copenhagen, Cochrane Collaboration) for the meta-analyses and with IBM SPSS Statistics for Macintosh, Version 30.0 (Armonk, NY: IBM Corp) for meta-regression and risk of publication bias assessments. All tests were two-sided with a *P*-level of *P* < 0.05 considered as statistically significant.

## Results

### Identification of studies

**Figure 1:**
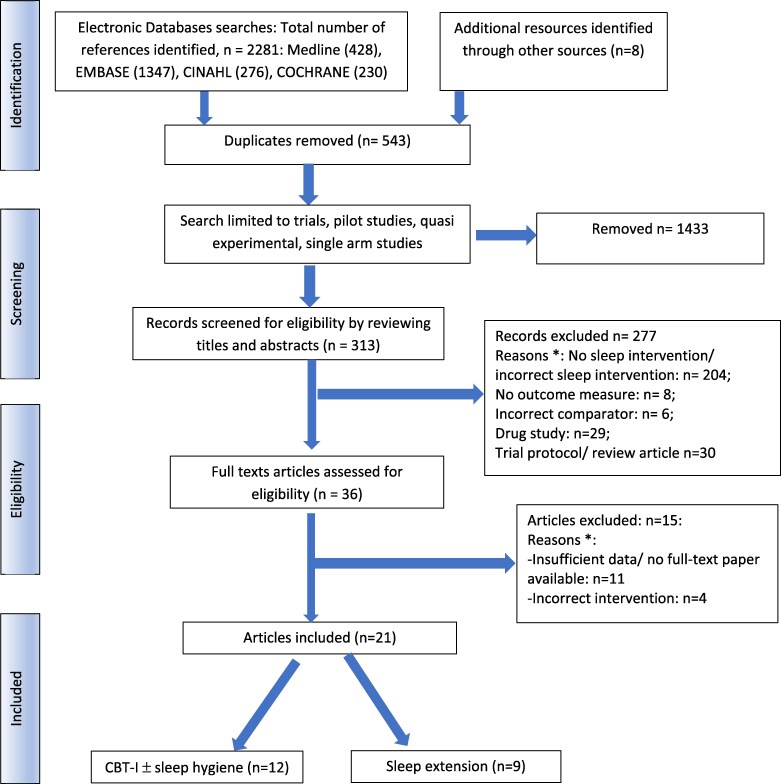
Flow chart to demonstrate the process of study selection. Key: CBT-I = cognitive behaviour therapy for insomnia, *n* = number. * For reasons excluded, there may have been more than 1 reason; however, the allocated category represents the first obvious reason for exclusion.

There were 313 abstracts derived from the search strategy, of which 35 met inclusion criteria for full text analysis; of these, 21 studies were included in the systematic review (*Figure 1,* including the reasons for exclusion; 23–43).

### Description of included studies and interventions

From the 21 studies that were published between 2011 and 2024, 12 used a CBT-I and/or sleep hygiene intervention (of which 11 consisted of CBT-I) and nine used a sleep extension intervention. *Table 1* describes the baseline characteristics of these studies. The studies originated from the Americas (9 studies in the USA, 1 in Canada), Europe (1 each from the UK, Ireland, Holland, and Germany), and Asia (3 in China, 2 in Japan, 1 each from Korea and Iran). The length of follow-up for studies ranged from 4 weeks to 12 months for CBT-I and/or sleep hygiene and from 2 weeks to 2.7 months for sleep extension.

**Table 1:** Baseline characteristics of included studies

					Intervention arm					Control arm				
A) Cognitive behaviour therapy for insomnia (CBT-I) or sleep education/hygiene														
First author, year, country, reference no	Population	Study type	Intervention (vs. control)	Study length	*n*	Age, years	Male %	BMI (SD)	Baseline measurements of outcomes + sleep variables ^b^	*n*	Age, years	Male %	BMI (SD)	Baseline measurements of outcomes + sleep variables ^b^
Amra, 2023, Iran,^22^	Insomnia	RCT	CBT-I single session (vs. routine care)	1 month	31	34.6 (9.5)	19.4	—	ISI 13.3 (3.7) HF 35.8 (21.5) LF 47.6 (19.01) LF: HF 2.3 (2.2)	26	36.6 (6.9)	34.6	—	ISI 13.3 (4.4) HF 43.9 (16.5) LF 39.6 (11.7) LF: HF 1.1 (0.9)
Groeneveld, 2024, Holland,^23^	Insomnia symptoms, T2DM	RCT	CBT-I (vs. control)	6 months	29	67.7 (8.4)	75.9	30.0 (7.0)	ISI 15.3 (3.7) TIB 8.4 (1.2) SLD (diary) 354 SLD (actigraph) 402 Efficiency 84.7% SBP 132.5 (17.8) DBP 75.5 (7.0)	28	71.8, (7.3)	57.1	29.9 (4.7)	ISI 13.1 (4.1) TIB 8.8 (1.4) SLD (diary) 378 SLD (actigraph) 444 Efficiency 86.9% SBP 138.8 (16.4) DBP 76.6 (7.4)
Ham, 2020, Korea,^24^	Insomnia with poor sleep quality	RCT	CBT-I sessions × 4 + sleep hygiene (vs. 1 session of group education)	12 months	24	53.83 (6.64)	0	24.42 (2.81)	ISI 11.29 (4.96). PSQI 8.46 (2.72) SBP 118.75 (11.37) DBP 70.88 (8.15)	20	55.45 (4.43)	0	23.91 (2.69)	ISI 12.85 (6.13) PSQI 8.60 (3.22) SBP 118.48 (13.06) DBP 70.23 (8.80)
Ito-Masui, 2023, Japan,^25^	Shift workers with SSD <6 h	Single-arm cross-over study	CBT-I, online for 4 weeks	4 weeks	61	32.9 (8.3)	18		PSQI 7.84 (2.46) SLD 332.4 (81.6) HR 74.79 (6.62)					
Jarrin, 2016, Canada,^26^	Insomnia for > 6 months	Single arm cross-over study	CBT-I over 6 weeks	6 weeks	65	51.8 (10.0)	33.8	—	ISI 17.0 (4.0) TST 347.0 (77.0) Efficiency 69.4% (15.5) HF (S2) 552.8 ms2 LF:HF (S2) 2.3 (1.5) REM HF 486.1 ms2 REM LF:HF 4.4 (4.2)					
Javaheri, 2020, USA,^27^	People with insomnia (ISI >10) + CVD	RCT	CBT-I, online, over 6 weeks (vs. sleep education)	6 weeks	15	70.3 (10.0)	61.1	30.1 (6.2)	ISI 15.6 (4.3) SBP 125.9 (20.0) DBP 65.5 (8.7)	14	72.9, (9.2)	87.5	27.6 (3.1)	ISI 15.6 (3.1) SBP 125.6 (14.2) DBP 68.5 (7.3)
Johann, 2020, Germany,^28^	Adults with insomnia disorder	RCT	CBT-I, face-to-face, 50 min session, once weekly × 8 weeks (vs. waiting list control)	8 weeks	23	40.8 (14.0)	39.1	—	ISI 15.4 (4.3) 24-h SBP 124 (11) 24-h DBP 83 (7) 24-h HR 75 (7) 24-h SDNN 50 (17) 24-h LF/HF 2.1 (0.5)	23	41.2 (15.1)	34.8	—	ISI 14.5 (5.0) 24-h SBP 124 (13) 24-h DBP 84 (5) 24-h HR 75 (9) 24-h SDNN 51 (19) 24-h LF/HF 2.1 (0.4)
Li, 2018, China,^29^	SSD <6 h, T2DM	RCT	2 × 30-min sleep education + T2DM education (vs. T2DM education only)	3 months	16	53.7 (17.2)	63	25.8 (3.2)	PSQI 7.6 (3.7) SBP 139.2 (3.1) DBP 89.0 (2.1)	15	51.6 (12.2)	60	26.1 (3.1)	PSQI 7.3 (4.1) SBP 138.7 (3.2) DBP 88.1 (2.2)
Liu, 2018, China,^30^	Elderly people with HTN + poor sleep quality	RCT	CBT-I + lifestyle intervention (vs. lifestyle intervention)	3 months	102	—	—	—	PSQI 14.17 (3.68) SBP 158.47 (13.36) DBP 87.15 (10.39)	82	—	—	—	PSQI 14.41 (4.07) SBP 156.27 (12.71) DBP 86.11 (10.03)
McGrath, 2017, Ireland,^31^	Mild sleep impairment + Raised BP	RCT	CBT-I, online, self-paced 6–8 weekly + lifestyle education (vs. same education alone)	8 weeks	54	59.7 (9.9)	40.3	27.0 (4.8)	PSQI 9.7 (3.7) ISI 13.0 (4.8) 24-h SBP 138.0 (11.7) 24-h DBP 83.5 (8.6)	67	58.3 (11.9)	37.3	27.1 (3.5)	PSQI 9.8 (3.8) ISI 12.0 (5.1) 24-h SBP 136.8 (10.8) 24-hour DBP 82.5 (7.7)
Palesh, 2019, USA,^32^	Breast cancer patients	RCT	Modified CBT-I for cancer pts: face-to-face × 2 session + telephone calls (vs. healthy eating control)	6 weeks	34	50.9 (7.9)	0	—	ISI Moderate 47% ISI severe 53% SDNN 17.93 (7.12) rMSSD 12.82 (4.58) HF 4.12 (0.75) LF/HF 3.40 (2.92) HR 87.06 (15.27)	37	53.8 (11.2)	0	—	ISI Moderate 55% ISI severe 45% SDNN 16.29 (6.48) rMSSD 10.28 (3.07) HF 3.75 (0.78) LF/HF 4.06 (2.73) HR 87.06 (15.27)
Yang, 2017, China,^33^	HTN	RCT	CBT-I, online, at least once/week for 8 weeks (vs. standard care)	8 weeks	53	56 (10)	40	24.27 (3.35)	ISI 11.82 (5.54) TST 249.59 (71.70) PSQI 13.12 (3.83) SBP 136.98 (10.61) DBP 81.41 (8.52)	53	57 (11)	38	24.36 (2.38)	ISI 10.52 (5.52) TST 256.2 (59.53) PSQI 12.10 (3.99) SBP 139.58 (16.2) DBP 81.08 (9.82)
**B) Sleep extension studies**														
Baron, 2019, USA,^34^	SSD < 7 h/ night + prehypertension/ stage 1 hypertension	RCT	Interactive smartphone app, brief coaching (vs. self-management group)	6 weeks	11	49.3 (14.4)	28.1 (6.4)	26.9	TST 402 (35.4) TIB 420 (37.2) ISI 9.33 (5.09) 24-h SBP 130.0 (11.8) 24-h DBP 81.67 (5.09)	5	37.8 (12.6)	100	27.2 (4.6)	TST 354 (72.6) TIB 412.8 (105) ISI 8.33 (8.05) 24-h SBP 122.0 (12.07) 24-h DBP 82.0 (11.06)
Cizza, 2014, USA,^35^	Obese (BMI 30–55), SSD < 6.5 h/night	RCT, but only prerandomization data considered, as a single arm	Only preintervention data analysed—RCT ended early before any intervention started	Median 2.7 months	125	40.6 (6.9)	24	38.4 (6.1	SLD (diary) 357.4 SLD (actigraph) 344.4 Efficiency 79.5% SBP 126.5 (12.3) DBP 75.1 (9.7)					
Gonzalez, 2024, USA,^36^	Short sleep duration	Single arm cross-over study	Acute sleep extension	2 weeks	22	60 (15)	27.3	25 (6)	SLD 405 (45) TIB 468 (52) Efficiency 86% SBP 123 (16) DBP 75 (8)					
Haack, 2014, USA,^37^	SSD < 7 h/ night + prehypertension/ stage 1 hypertension	RCT	Advised to increase TIB by 1 h/ night, sleep hygiene advice (vs. sleep hygiene)	6 weeks	13	46.9 (12.3)	46.1	26.5 (1.2)	SLD 378 min (12) Beat-to-beat SBP 142 (4) Beat-to-beat DBP 82 (3)	9	48.4 (12.3)	22.2	26.0 (1.2)	SLD 372 min (18) Beat-to-beat SBP 146 (7) Beat-to-beat DBP 84 (3)
Hartescu, 2021, UK,^38^	SSD < 6.5 h/night	RCT	Advised to increase TIB by 1 h/ night, Counselling sessions of CBT principles	6 weeks	10	39.9 (8.8)	100	29.3 (1.6)	PSQI 10.8 SLD 344.2 min TIB 398.4 min Efficiency 85.8% SBP 136 (10) DBP 84 (10)	8	43.0 (10.1)	100	29.8 (4.2)	PSQI 9.44 SLD 349 min TIB 431.6 Efficiency 80.7% SBP 135 (9) DBP 84 (9)
Kubo, 2011, Japan,^39^	Daytime workers with SSD < 6 h	cross-over study	Increase TIB at weekends to > 8 h (or habitual sleep at weekend)	3 weeks	26	38.3 (8.1)	69.2	—	—					
Matthew, 2024, USA,^40^	College students >18 years with SSD	Single arm cross-over study	Advised to increase TIB by 1 h/ night	1 week	11	20.3 (1.5)	17	24.5 (3.5)	SLD 6.93 (0.83) ESS 8.08 (4.17) SBP 116.18 (12.45) DBP 66.36 (8.05) HR 73.91 (8.67)					
Reynold, 2014, USA,^41^	Sleep duration 6–9 h, mean age 31.7, 28.6% male, BMI <30	RCT	Increase TIB by 3 h/ night (vs. control)	1 week	8	—	—	—	TIB (acti) 476 (62) TST (acti) 407 (35) ESS 4.50 (3.42) PSQI 3.88 (0.99) SBP 112.19 (9.28) DBP 71.56 (8.62) HR 75.38 (16.62)	6	—	—	—	TIB (acti) 483 (44) TST (acti) 417 (21) ESS 4.67 (2.73) PSQI 3.0 (1.9) SBP 115.56 (11.52) DBP 70.28 (8.13) HR 78.11 (9.93)
Stock, 2020, USA,^42^	SSD <7 h	Single arm cross-over study	Advised to increase TIB by 1 h/ night	1 week	53	20.5 (1.1)	30	—	SLD (24hr) 456.3 (38.6) Efficiency 91.0 (2.6) SBP 117.1 (19.1) DBP 69.9 (10.4) HR 80.0 (13.4)					

Continuous variables represented as mean (SD) or median (IQR). Pre and poststudies have similar baseline characteristics in intervention and control arms. ^b^ Units for variables: All BP measurements in mmHg; SLD, TIB, and TST in minutes; HR measured as beats per minute; SDNN in milliseconds; HF and LF in milliseconds (presented as normalized unit where available). **Abbreviations:** BMI, body mass index, CBT-I, Cognitive Behaviour Therapy for Insomnia, DBP, diastolic blood pressure, HR, heart rate, HRV, heart rate variability, IQR, interquartile range, ISI, insomnia severity index score, min, minutes, LF, low frequency, LF:HF ratio, low-frequency: high-frequency power ratio, *N*, number, PSQI, Pittsburgh Sleep Quality Index global score, RCT, randomized controlled trial, REM, rapid eye movement phase of sleep, S2, second stage of sleep, SBP, systolic blood pressure, SD, standard deviation, SDNN, standard deviation of normal-to-normal intervals. SLD, sleep duration, SSD, short sleep duration, TIB, time in bed, TST, total sleep time.

### Description of study participants characteristics

Considering the baseline characteristics for the total cohort, the mean age was 46.7 years (SD 14.1) and BMI was 28.9 kg/m^2^ (3.9), with a percentage of males of 28.5%. The mean systolic and diastolic blood pressures were 134.8 (SD 11.7) and 79.2 (7.1) mmHg, respectively, suggesting most people did not have hypertension at baseline. Regarding sleep variables, the mean levels for ISI, PSQI global score, sleep duration, and time-in-bed were 13.3 (2.4), 10.9 (3.1), 382.6 (43.9) min, and 467.2 (49.0) min, respectively.

### Description of assessment of study quality

From the 19 studies considered for study quality, only seven studies had low concern throughout all assessment categories, with most other studies having at least some concern, Supplementary material online, *Appendix B*. Two studies (Liu, Yang) had abstracts and tables written in English, but the methods were written in Chinese and could not be translated. We took assistance via email from the first author of a separate systematic review (Dr. Kimberly Savin, San Diego State University, USA) that translated Yang et al. previously to aid quality assessment, but we were unable to perform this for Liu et al., hence it was not assessed for quality.

### Description of outcomes

#### Effect of sleep interventions on sleep variables

**Figure 2:**
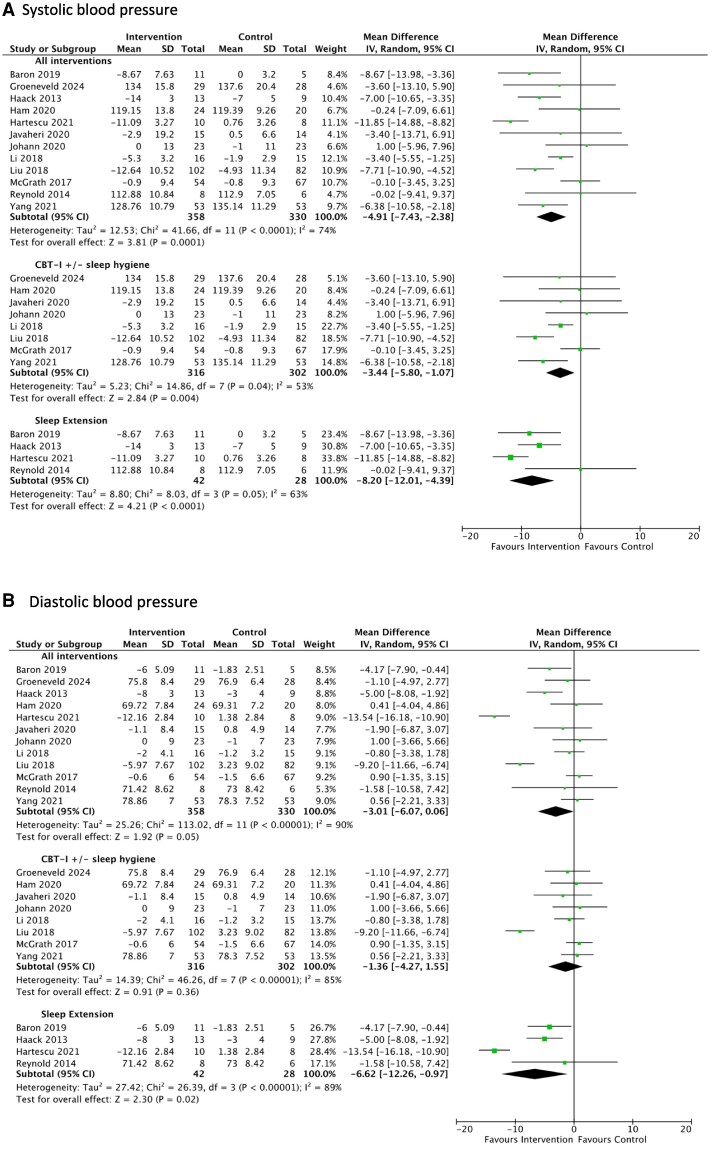
Forrest plots for the results of the meta-analysis assessing the effect of sleep interventions in RCTs only, on levels of (*A*) systolic blood pressure and (*B*) diastolic blood pressure in (i) all interventions, (ii) Cognitive Behavioural Therapy for Insomnia (CBT-I) and/or sleep hygiene, and (iii) sleep extension. Key: CI = confidence interval, CBT-I = Cognitive Behavioural Therapy for Insomnia, I^2^= heterogeneity test, IV = inverse variance, RCT = randomized controlled trial, SD = standard deviation. All blood pressure variables are measured in mmHg. The test for subgroup differences between (1) CBT-I and/or sleep hygiene and (2) sleep extension interventions was significant for systolic (*P* = 0.04) but not diastolic blood pressure (*P* = 0.10).

**Figure 3:**
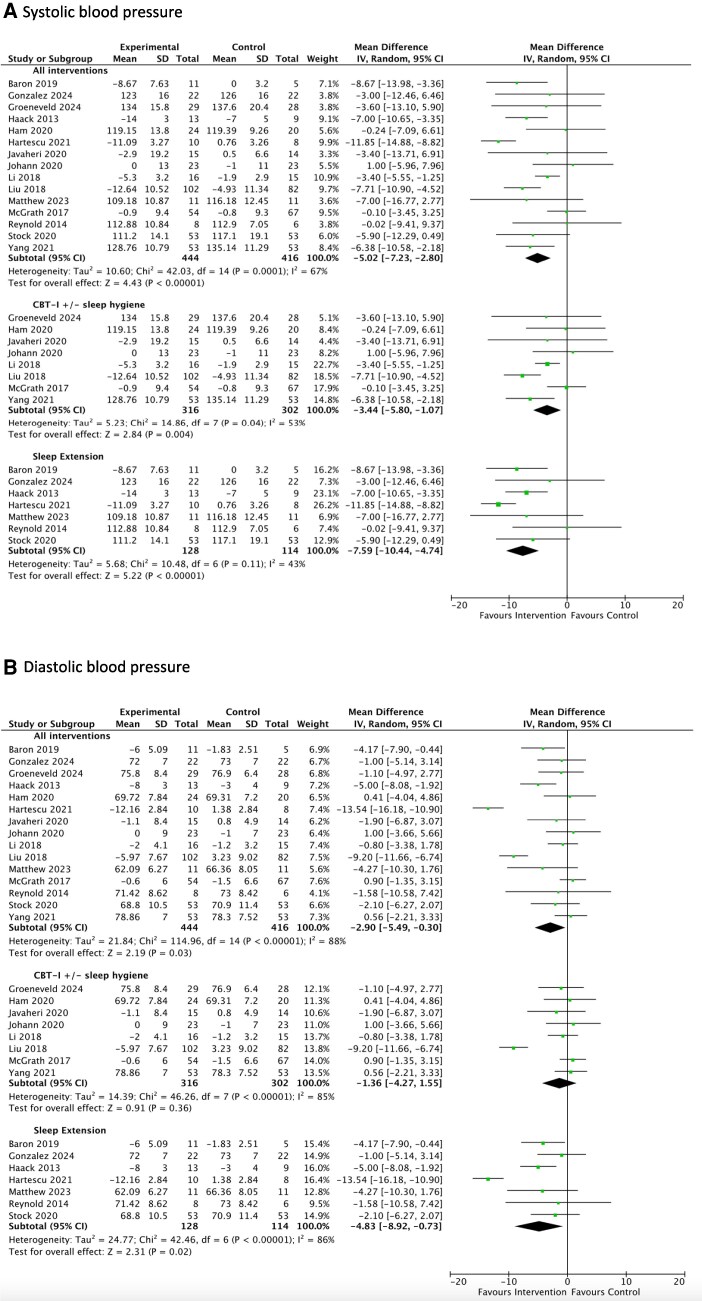
Forrest plots for the results of the meta-analysis assessing the effect of sleep interventions in combined RCTs and non-RCTs on levels of (*A*) systolic blood pressure and (*B*) diastolic blood pressure in (i) all interventions, (ii) Cognitive Behavioural Therapy for Insomnia (CBT-I) and/or sleep hygiene, and (iii) sleep extension. Key: CI = confidence interval, CBT-I = Cognitive Behavioural Therapy for Insomnia, *I*^2^= heterogeneity test, IV = inverse variance, RCT = randomized controlled trial, SD = standard deviation. All blood pressure variables are measured in mmHg. The test for subgroup differences between (1) CBT-I and/ or sleep hygiene and (2) sleep extension interventions was significant for systolic (*P* = 0.03) but not diastolic blood pressure (*P* = 0.18).

**Figure 4:**
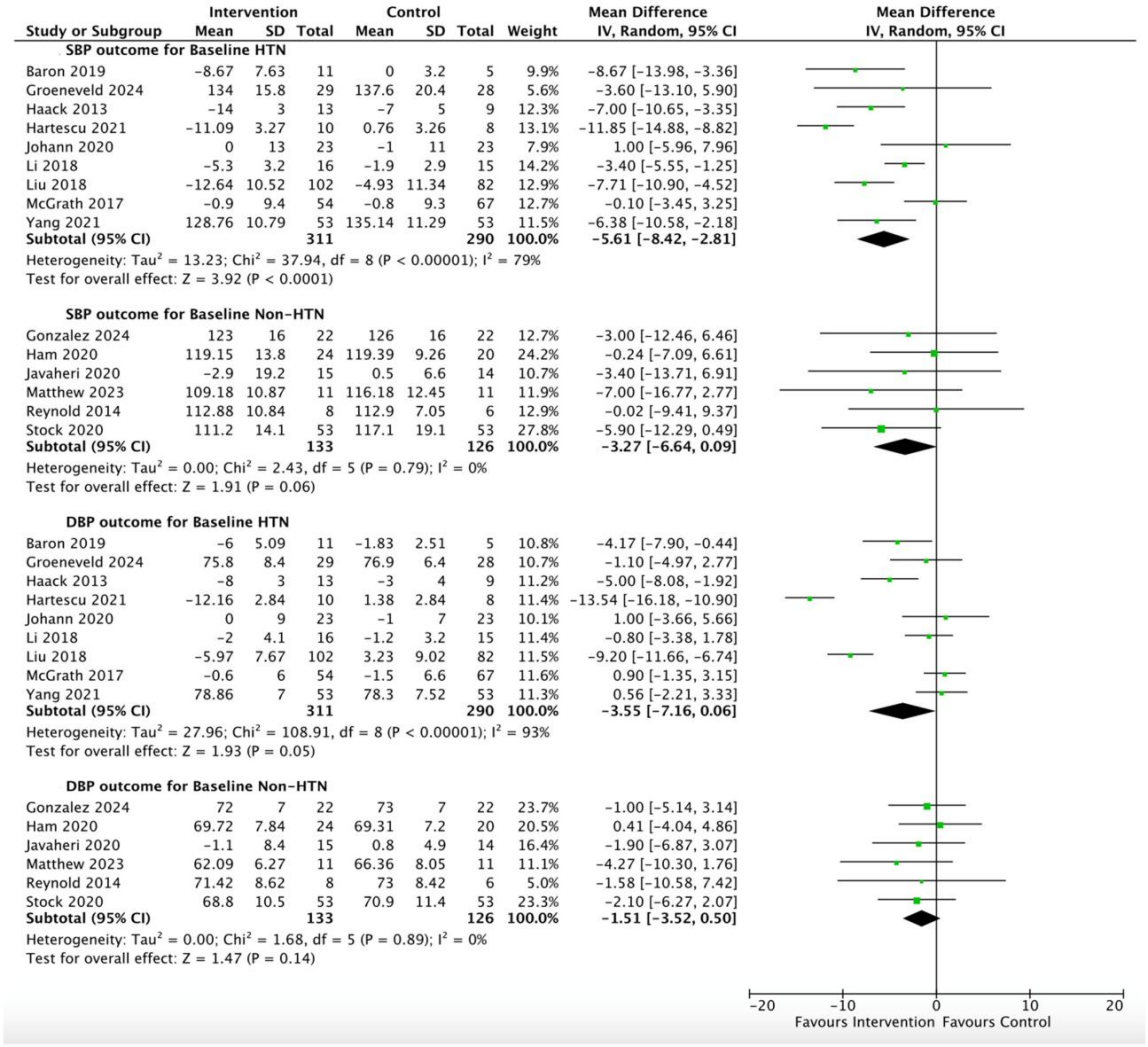
Forrest plots for the results of the meta-analysis assessing the effect of sleep interventions on levels of systolic and diastolic blood pressure according to whether studies were categorized as having baseline hypertension or nonhypertension. Key: CI = confidence interval, CBT-I = Cognitive Behavioural Therapy for Insomnia, DBP = diastolic blood pressure, HTN = hypertension, *I*^2^= heterogeneity test, IV = inverse variance, non-HTN = non- hypertension, SBP = systolic blood pressure, SD = standard deviation. All blood pressure variables are measured in mmHg. The hypertension category refers to a combination of ‘stage 1 or 2 hypertension’ and the non-hypertension category refers to a combination of ‘normal or elevated BP’ (according to 2017 ACC/AHA guidelines).

Data from 19 of the 21 studies were extracted to conduct meta-analyses (*Figures 2–4*, Supplementary material online, *Appendix C–G*, *Graphical Abstract*). Use of sleep interventions significantly improved sleep variables, with reductions for ISI and PSQI global score averaging 4.37 points (95% CI 2.21, 6.52, *P* < 0.0001, *I*^2^ for heterogeneity 88%) and 1.52 (0.75, 2.29, *P* = 0.0001, *I*^2^ 62%), respectively, compared to control. Improvements in sleep duration and TIB averaged 49.59 min (20.49, 78.69, *P* = 0.0008, *I*^2^ 94%) and 85.75 min (5.41, 166.09, *P* = 0.04, *I*^2^ 62%), respectively, compared to control.

#### Effect of sleep interventions on blood pressure outcomes

From 15 studies, of which 12 were RCTs, there were significant reductions in levels of systolic blood pressure observed in six studies (Barron, Haack, Hartescu, Li, Liu, and Yang) and diastolic blood pressure in four studies (Barron, Haack, Hartescu, and Liu). Two studies that were included in the systematic review, but not the meta-analysis, presented interesting findings. The randomized controlled trial of Cizza et al., was terminated early, as within the 2.7 months between baseline and screening, 125 participants significantly increased their sleep duration, on average by 31 min, without undertaking any intervention, which led to significant reductions in levels of systolic blood pressure, 126.5 mmHg (SD 12.3) and 124.3 (12.2), respectively, *P* = 0.02, but not diastolic blood pressure.^35^ Secondly, Kubo et al. conducted a sleep extension intervention of increasing time-in-bed to ≥8 h at weekends, which increased total sleep time increased (*P* < 0.05) but did not change blood pressure after 3 weeks (exact values were not provided in the study).^39^

##### Meta-analysis of RCTs

Considering all sleep interventions, from 12 studies (*n* = 688), use of the intervention was associated with a significant reduction in systolic blood pressure levels averaging 4.91 mmHg (95% CI 2.38, 7.43, *P* < 0.00001, I^2^ = 74%), compared to control, *Figure 2*. On further analysis, within eight studies (*n* = 618) of CBT-I and/or sleep hygiene interventions, systolic blood pressure levels decreased significantly, averaging 3.44 mmHg (1.07, 5.80; *P* = 0.004, *I*^2^ = 53%) compared to control. In four studies (*n* = 70) of sleep extension interventions, systolic blood pressure reduction was significant, averaging 8.20 mmHg (4.39, 12.01; *P* < 0.00001, I^2^ = 63%).

For diastolic blood pressure outcomes, the average reductions were not significant within all interventions (*P* = 0.05) or within CBT-I and/or sleep hygiene studies (*P* = 0.36). However, use of sleep extension in four studies (*n* = 70) led to significant reductions averaging 6.62 mmHg (0.97, 12.26, *P* = 0.02, *I*^2^ = 89%) compared to control. Heterogeneity was considered high for diastolic blood pressure analyses. The test for subgroup differences between (i) CBT-I and/or sleep hygiene and (ii) sleep extension interventions was significant for systolic (*P* = 0.04) but not diastolic blood pressure (*P* = 0.10), *Figure 2*.

##### Meta-analysis of non-RCTs

Within three studies of sleep extension (*n* = 172), use of the intervention, led to significant reductions in systolic blood pressure, averaging 5.45 mmHg (0.79, 10.10, *P* = 0.02, I^2^ = 0%), but were not significant for diastolic blood pressure (*P* = 0.12), Supplementary material online, *Appendix D*.

##### Meta-analysis of combined RCTs and non-RCTs

Considering all sleep interventions, from 15 studies (*n* = 860, mean duration 10.3 weeks), use of the intervention was associated with a significant reduction in systolic blood pressure levels averaging 5.02 mmHg (95% CI 2.80, 7.23) *P* < 0.00001, *I*^2^ = 67%, compared to control, *Figure 3*. In seven studies (*n* = 242, mean duration 3.3 weeks) of sleep extension interventions, systolic blood pressure reduction was significant, averaging 7.59 mmHg (4.74, 10.44; *P* < 0.00001, *I*^2^ = 43%). CBT-I and/or sleep hygiene studies (mean duration 16.5 weeks) were RCTs only and are described above. For diastolic blood pressure outcomes, there were significant reductions averaging 2.90 mmHg (0.30, 5.49; *P* = 0.03, *I*^2^ = 88%) and 4.83 mmHg (0.73, 8.92; *P* = 0.02, *I*^2^ = 86%) in all interventions and sleep extension interventions, respectively, compared to control. The test for subgroup differences between (i) CBT-I and/or sleep hygiene and (ii) sleep extension interventions was significant for systolic (*P* = 0.03) but not diastolic blood pressure (*P* = 0.18), *Figure 3*.

##### Sub-group analysis

Studies with mean blood pressure levels in the categories of stage 1 and 2 hypertension at baseline demonstrated the highest reductions in systolic blood pressure, averaging 4.98 mmHg (1.28, 8.67, *I*^2^ = 83%, *P* = 0.008, *n* = 395 from seven studies) and 7.40 mmHg (5.00, 9.81, *I*^2^ = 0%, Tau-squared 0.0, *P* < 0.00001, *n* = 206 from two studies), *Table 2*. The two remaining categories, which consisted of lower numbers of participants, demonstrated nonsignificant reductions in systolic blood pressure: elevated blood pressure, 3.18 mmHg (−3.79, 10.15, *I*^2^ = 0%, *P* = 0.37, *n* = 73) and normal blood pressure, 3.30 mmHg (−0.55, 7.15, *I*^2^ = 0%, *P* = 0.09, *n* = 186). When these latter two categories were combined (i.e. participants without hypertension), the average systolic blood pressure reduction just missed significance, 3.27 mmHg (−0.09, 7.15, *I*^2^ = 0%, *P* = 0.06, *n* = 259 from six studies), *Figure 4*. Considering the same analysis for the diastolic blood pressure outcome, the average reduction was only significant in stage 2 hypertension, 7.21 (3.10, 11.32, *I*^2^ = 0%, *P* = 0.0006).

**Table 2:** Pooled changes in systolic and diastolic blood pressure according to baseline blood pressure category of individual studies

			SBP (mmHg)			DBP (mmHg)		
Blood pressure category (mmHg)	Number of studies	Number of people	Post intervention change (95% CI)	*P*-value	I^2^ (%)	Post intervention change (95% CI)	*P*-value	I^2^ (%)
Normal: SBP <120 and DBP <80	4	186	−3.30 (−7.15, 0.55)	0.09	0	−1.60 (−4.20, 1.00)	0.23	0
Elevated: SBP 120–129 and DBP <80	2	73	−3.18 (−10.15, 3.79)	0.37	0	−1.37 (−4.55, 1.81)	0.40	0
Stage 1 HTN: SBP 130–139 or DBP 80–90	7	395	−4.98 (−8.67, −1.28)	**0.008**	83	−2.50 (−6.80, 1.81)	0.26	93
Stage 2 HTN: SBP≥140 or DBP≥90	2	206	−7.40 (−9.81, −5.00)	**<0.00001**	0	−7.21 (−11.32, −3.10)	**0.0006**	77
No hypertension (normal + elevated combined)	6	259	−3.27 (−6.64, 0.09)	0.06	0	−1.51 (−3.52, 0.50)	0.14	0
Hypertension (Stages 1 + 2 combined)	9	601	−5.61 (−2.81, −8.42)	**<0.00001**	79	−3.55 (−7.16, 0.06)	0.05	93

A total of 15 studies (*n* = 860 people) were placed into categories based on the baseline mean blood pressure levels. As per 2017 ACC/AHA guidelines (reference ^19^), if the baseline SBP and DBP were in two categories, the study was allocated to the higher BP category. Abbreviations: 95% CI, 95% confidence intervals, DBP, diastolic blood pressure, HTN, hypertension, *I*^2^, *I*^2^ heterogeneity statistic, mmHg, millimetres of Mercury, *N*, number, SBP, systolic blood pressure.

Significant *P*-values have been highlighted in bold.

##### Sensitivity analyses for blood pressure outcomes

First, for the risk of publication bias, eleven studies with less than 60 participants were removed before re-performing the analysis. Within the remaining four studies (McGrath, Stock, Yang and Liu) with 517 participants, use of the intervention significantly reduced systolic blood pressure reduction, averaging 4.91 mmHg (0.96, 8.86, *I*^2^ = 74%, *P* = 0.01) compared to control, but was not significant for the diastolic blood pressure reduction (*P* = 0.35), Supplementary material online, *Appendix E*. Visual inspection of funnel plots did not reveal asymmetry, Supplementary material online, *Appendix E*. Secondly, when the meta-analysis results were performed according to the risk of bias categories, the test for subgroup differences between categories was not significant for either systolic (*P* = 0.20) or diastolic (*P* = 0.08) blood pressure, Supplementary material online, *Appendix I*. Furthermore, the *I*^2^ and Tau-squared values for the lowest risk of bias category were 14% and 1.79 for systolic and 26% and 1.48 for diastolic blood pressure, respectively, which were some of the lowest heterogeneity levels observed in this overall analysis.

##### Meta-regression analysis for blood pressure outcomes

Meta-regression models demonstrated the type of intervention to be a significant confounder for the outcome of systolic blood pressure (*P* = 0.026), *Table 3*. Specifically, studies using CBT-I and/or sleep hygiene were found to have a smaller effect size than those using sleep extension, by a mean of 4.2 mmHg (95% CI 0.5, 7.9, *P* = 0.026, *Figure 2*). A similar trend was observed for the outcome of diastolic blood pressure, although this did not reach statistical significance (*P* = 0.147). Of the demographic factors considered, studies with a larger proportion of male participants were found to have a significantly larger effect size. Specifically, the benefit of the intervention on systolic blood pressure increased by 0.9 mmHg (95% CI: 0.1, 1.7, *P* = 0.021) per 10 percentage point increase in the proportion of males, with a similar increase of 1.0 mmHg (0.2, 1.8, *P* = 0.011) observed for diastolic blood pressure, Supplementary material online, *Appendix H*. This effect was largely driven by two single-sex studies, specifically Ham et al., which only included females and reported no effect, in contrast to Hartescu et al., which only included males and observed the largest effect.^24,38^ When these two studies were removed from the analysis, the overall effects on systolic and diastolic blood pressure were no longer significant, *P* = 0.93 and *P* = 0.85, respectively.

**Table 3:** Meta-regression analysis of the effects of sleep interventions on blood pressure levels

		SBP (mmHg)		DBP (mmHg)	
Variable	*N* studies^a^	Coefficient (95% CI)	*P*-value	Coefficient (95% CI)	*P*-value
Age (per decade)	14	0.9 (−0.8, 2.6)	0.314	0.8 (−0.8, 2.5)	0.324
BMI (per 2 kg/m^2^)	11	−1.6 (−4.3, 1.1)	0.248	−2.0 (−4.6, 0.6)	0.136
Proportion of males (per 10 percentage point)^b^	14	−0.9 (−1.7, −0.1)	**0.021**	−1.0 (−1.8, −0.2)	**0.011**
Geographical location (North America vs. Other)	15	−1.1 (−5.8, 3.7)	0.663	−0.1 (−5.5, 5.3)	0.973
Intervention (CBT-I/sleep hygiene vs. extension)	15	4.2 (0.5, 7.9)	**0.026**	3.6 (−1.3, 8.4)	0.147
Baseline SBP (per 10 mmHg)	15	−1.1 (−3.0, 0.8)	0.245	—	—
Baseline DBP (per 10 mmHg)	15	—	—	−1.5 (−5.2, 2.2)	0.423
Baseline hypertension (Stage 1/2 vs. normal/elevated)	15	−3.6 (−8.5, 1.3)	0.150	−0.4 (−7.9, 3.0)	0.382
Intervention duration (per month)	15	0.4 (−0.3, 1.2)	0.276	0.3 (−0.5, 1.1)	0.496
Year of publication (per decade)	15	−1.1 (−4.7, 2.6)	0.565	−0.6 (−5.3, 4.2)	0.819
Study design (RCT vs. other)	15	−0.4 (−7.0, 6.2)	0.896	0.7 (−6.1, 7.4)	0.849
Intervention delivery (face-to-face vs. remote/digital)	13	−1.1 (−6.7, 4.5)	0.695	−1.7 (−8.0, 4.6)	0.605
BP measurement (single vs. average 24-h)	15	3.2 (−1.9, 8.3)	0.221	2.72 (−3.6, 9.0)	0.395

Results are from univariable meta-regression analyses, with separate models produced for each variable. The dependent variable was the mean difference in BP between the intervention and control groups; negative values indicate a lower BP in the intervention arm. For binary variables, the coefficient represents the difference in the effect size between the two subgroups of studies. For continuous variables, the coefficient represents the change in the effect size per the stated number of units increase in the factor. Bold *P*-values are significant at *P* < 0.05. ^a^The number of studies included in the analysis, after excluding those with missing data for the variable of interest. ^b^The coefficient represents the change in the effect size per 10 percentage point increase in the proportion of males in the study (e.g. for a study comprising 40% vs. 30% males).

**Abbreviation:** 95% CI, 95% confidence intervals, BP, blood pressure, CBT-I, Cognitive Behaviour Therapy for Insomnia, DBP, diastolic blood pressure, *N*, number, RCT, randomized controlled trial, SBP, systolic blood pressure.

#### Effect of sleep interventions on heart rate

From the eight studies reporting heart rate, only one study (Palesh et al.) reported a significant difference between intervention and control arms.^32^ Within this specific cohort of females undergoing treatment for breast cancer, after undertaking CBT-I, there was a lower increase in mean heart rate in the intervention arm, 0.18 beats per minute (9.71), compared to 8.26 beats per minute (SD 12.89) in the control arm, *P* < 0.01. For the meta-analysis, within eight studies (*n* = 509) of all sleep interventions, there was a non-significant reduction averaging 1.36 beats per minute (−0.46, 3.18; *P* = 0.14, I^2^ = 47%) compared to control, Supplementary material online, *Appendix F*. However, within four sleep extension studies (*n* = 164), the reduction in heart rate was significant, averaging 1.24 beats per minute (0.44, 2.44; *P* = 0.04, *I*^2^ = 0%), but remained not significant for four studies of CBT-I and/or sleep hygiene (*n* = 345), 1.04 beats per minute (−2.42, 4.49; *P* = 0.56, *I*^2^ = 71%).

#### Effect of sleep interventions on heart rate variability

Four studies reported heart rate variability indices, all of which consisted of a CBT-I and/or sleep hygiene intervention. Palesh et al. were the only study to report any significant changes, with a change from baseline to follow-up in mean HF power of 0.9 milliseconds (SD 0.83) compared to 1.04 (1.17) and mean SDNN of 0.41 milliseconds (6.6) compared to −4.76 (4.78) in the intervention and control arms, respectively (both *P* < 0.01).^32^ Within the meta-analyses, there were no differences between intervention and control for LF: HF power ratio, HF power, and SDNN, Supplementary material online, *Appendix G*.

## Discussion

This systematic review and meta-analyses is the first to demonstrate that using behavioural sleep interventions in people with poor sleep health, but without OSA, leads to clinically significant reductions in levels of systolic and diastolic blood pressure, averaging 5.0 and 2.9 mmHg, respectively. The heterogeneity, however, was moderate and high for systolic and diastolic blood pressure outcomes, which can affect the generalizability of the findings, and was only partially explained through meta-regression analysis. Individually, the interventions of (i) CBT-I and/or sleep hygiene and (ii) sleep extension both significantly decreased systolic blood pressure, but only sleep extension decreased diastolic blood pressure. The highest reductions in systolic blood pressure levels were observed in stage 1 and 2 hypertension categories, averaging 4.9 and 7.4 mmHg, respectively, upon improving sleep, while the two nonhypertension categories were not associated with a significant reduction. This suggests a differential impact according to baseline blood pressure. Finally, sleep extension interventions decreased heart rate by an average of 1.24 beats per minute, but there were no other differences observed for heart rate or heart rate variability indices in any sleep intervention.

Despite CBT-I and/or sleep hygiene interventions improving insomnia symptoms and sleep quality by 4.37 and 1.52 index points, respectively, on average, there was no change in diastolic blood pressure, heart rate, or heart rate variability. Epidemiological studies associate insomnia and poor sleep health with more adverse levels of these risk factors; however, our results suggest that this sleep intervention alone does not improve the risk factor level. This may represent a true effect or perhaps should be considered within the context of other factors, such as lower participant numbers reflecting underpowering, particularly for heart rate and heart variability outcomes. In contrast, extending sleep duration by an average of 49 min improved all outcomes.

The potential sources of heterogeneity for systolic and diastolic blood pressure outcomes were explored through meta-regression models, but could only be partially explained by the differences in sex ratios across the studies and the type of sleep intervention for systolic blood pressure only. This suggests some residual heterogeneity is unaccounted for. Sleep interventions appeared to be more effective in reducing blood pressure in males; however, this was mainly influenced by two single-sex studies and when these two studies were removed, the effect was no longer significant. For now, this remains an area of interest.

To help explain our results, we should consider the plausible mechanisms associating changes in sleep and blood pressure. Insufficient sleep increases various hormone levels, including angiotensin II and aldosterone, via sympathetic nervous system activation in the renin-angiotensin system.^3^ This results in increased vasoconstriction with arterial stiffness, systemic inflammation, and endothelial dysfunction favouring an increase in blood pressure. Aldosterone also increases through dysregulation of the hypothalamic-pituitary-adrenal axis mediated through increased adrenocorticotropic hormone release. Improving insufficient sleep through using behavioural interventions decreases the sympathetic activation favouring a lowering of blood pressure. The autonomic nervous system also plays an important role in controlling heart rate and heart rate variability.^43^ Insomnia is associated with decreased heart rate variability; however, we found use of CBT-I interventions did not change heart rate variability indices based on four studies.

Meta-analyses have demonstrated that people with poor sleep health generally have higher systolic and diastolic blood pressure levels, averaging 4.37 and 1.25 mmHg, respectively, compared to people who sleep well, albeit not meeting statistical significance.^4^ When viewed within the context of the present results, it could be suggested that there may be potential to reverse this using behavioural sleep interventions. Furthermore, the effect sizes observed for systolic and diastolic blood pressure average reductions, from the present study, are larger than those reported in meta-analyses of (a) CPAP intervention in people with OSA (2.6 and 2.0 mmHg, respectively), (b) changing work schedules for rotating and night-shift workers (0.26 and 0.06 mmHg), and (c) general CBT intervention used in the general population (0.65 and 0.78 mmHg) or in people with T2DM (1.8 and 2.88 mmHg).^10,44–46^ Finally, the average systolic blood pressure reduction of 5.0 mmHg derived from the present study is comparable to a large-scale meta-analysis of pharmacological blood pressure lowering medications, which reported that a 5 mmHg reduction of systolic blood pressure was associated with decreased risk of CVD events by 10% after four years.^47^ It is not known, however, if behavioural sleep interventions are able to sustain systolic blood pressure reductions over the long-term, as the average length of studies analysed was 16.5 and 3.3 weeks for CBT-I and/or sleep hygiene and sleep extension, respectively. That said, when the long-term effects of a CBT-I intervention were analysed for its impact on glycaemia, one study reported a sustained legacy effect of reducing fasting glucose levels after 6.2 years.^48^

Regarding limitations of the study, the quality of methods used in studies was variable, as demonstrated in the study quality analysis. Some studies had relatively smaller sample sizes where blood pressure or heart rate may not have been the main outcome variable. Secondly, we included various subclinical dimensions of sleep health for the definition of poor sleep health, as they tend to overlap (e.g. concomitant insomnia and short sleep duration). Thirdly, there was also some variability in how the outcomes were measured, where some studies used 24-hour averages for blood pressure and heart rate, while other studies used a single reading, but all studies used automated measurements. Despite these differences, however, the studies showed consistent trends in outcomes, as demonstrated by the meta-analyses, particularly for blood pressure. Fourthly, most studies did not account for potential changes in medications, dosing regimens, or adherence levels during the trials, which are possible confounders. Within the four studies with the longest intervention durations of 3–12 months, only Ham et al., lasting 12 months, featured a normotensive population at baseline.^24^ As the remaining 11 studies were conducted over limited time periods, however, it is likely that medications may not have been changed from baseline. For example, McGrath et al. reported that only 4.5% of participants had their antihypertensive medication doses changed.^31^ In contrast, Haack et al. discontinued participants who needed a change in medication or dosage during their trial.^37^ Furthermore, only 2 of the 15 studies had participants with stage 2 hypertension (SBP ≥140 or DBP ≥90 mmHg) at baseline. Fifthly, for the sleep interventions, we combined CBT-I and/or sleep hygiene, as there tends to be some overlap in the contents of these interventions and then combined all interventions for an overall outcome. However, the results of the meta-analyses demonstrated that the heterogeneity levels for blood pressure outcomes were similar whether they were grouped together or investigated separately. Finally, on assessing the outcomes within the meta-analyses, we found some studies reported the data as the final postintervention value, while other studies reported the mean change in the variable. However, this was observed in similar meta-analyses.^11,13,14,16^

The strengths of this study are the systematic approach and robust methods used to obtain our results, ensuring they met accepted standards for meta-analyses. We considered a wide range of sleep behaviours as causes for insufficient sleep, as well as for blood pressure and heart rate-related terms, which were used in the comprehensive list of search terms in the search strategy. We analysed full-text articles and abstracts for studies in all languages, allowing us to detect as many eligible studies as possible. The studies incorporated a broad range of adult age, sex, sleep behaviours, and baseline blood pressure levels, suggesting the results could be applicable to a wide variety of people.

### Potential implications for clinical practice

A recent meta-analysis demonstrated use of CBT-I and/or sleep hygiene interventions decreases glycated haemoglobin, HbA1c, level by a clinically significant 0.43% in people with T2DM, while sleep extension improved some early risk markers of glucose metabolism.^11^ The present meta-analysis provides preliminary evidence that the same sleep interventions can reduce systolic blood pressure by a clinically significant 5 mmHg, which is associated with an approximate 10% reduction in CVD over four years.^47^ Given the impact of these behavioural sleep interventions on these important cardiovascular risk factors, it could be proposed that detecting individuals with poor sleep health, followed by implementing interventions to improve their sleep, may be a cost-effective approach to improving CVD risk. However, future research in large-scale trials is still needed, particularly to determine if blood pressure reductions can be sustained in the long-term. Assessing for forms of poor sleep health can be performed through commonly used sleep assessment tools, including the Insomnia Score Index and Pittsburgh Sleep Quality Index, while many modern-day smart wristwatch devices can assess sleep tracking for measurements.^49^

Recent international guidelines for the management of hypertension, chronic coronary syndrome, and atrial fibrillation include management of sleep and sleep disorders; however, they focus almost exclusively on treatment strategies for OSA.^50^ The present meta-analysis provides preliminary evidence beyond OSA, in treating people with poor sleep health who are estimated to represent 30–40% of adult populations.^1,2^

As the burden of CVD and its precursor risk factors remains a public health concern and as some newer pharmacological therapies for reducing CVD risk may have cost implications, improving lifestyle risk factors could be a useful strategy going forward. Lifestyle advice and recommendations traditionally focus on modification of diet and physical activity; however, improving sleep could emerge as a useful adjunct, especially as the former behavioural changes cannot always be achieved or maintained in the long-term.

In conclusion, the use of behavioural sleep interventions led to clinically significant reductions in systolic and diastolic blood pressure in people with poor sleep health. As both sleep interventions are feasible and efficacious in pilot studies, future research should examine the value of using behavioural sleep interventions, with standardized sleep protocols, in poor sleep health in large scale, well-designed RCTs for long-term outcomes, as a potential target to aid current measures of preventing CVD.

## Supplementary Material

oeag006_Supplementary_Data

## Data Availability

Data used in this study (including an unpublished protocol) is retained by the research team.
