# Supplementary material for: Effect of behavioural sleep interventions on blood pressure, heart rate, and heart rate variability in adults with poor sleep health: a systematic review, meta-analysis, and meta-regression analysis
Source: Eur Heart J Open. 2026 Jan 20;6(1):oeag006. doi: 10.1093/ehjopen/oeag006 (PMC12915584; doi:10.1093/ehjopen/oeag006)
Supplement: oeag006_Supplementary_Data [file oeag006_supplementary_data.zip › Supplementary-material.pdf]

Supplementary material:

Effect of behavioural sleep interventions on blood pressure, heart rate and heart rate variability in adults with poor sleep health: a systematic review, meta-analysis and meta-regression analysis.

Samiul A Mostafa, Wasim Hanif, George Balanos, Krishnarajah Nirantharakumar, Jason G. Ellis, Abd A Tahrani

All authors: University of Birmingham, Birmingham, UK, except Professor Ellis: University of Northumbria, UK. Correspondence to Dr. Samiul A Mostafa. Email: s.a.mostafa@bham.ac.uk and samiul.mostafa@uhb.nhs.uk

CONTENTS:

Appendix A. Database search strategies

Appendix B. Risk of bias assessments

Appendix C. Forrest plots: effect of sleep interventions on sleep variables

Appendix D. Forrest plots: effect of sleep interventions on blood pressure in non-RCTs

Appendix E. Sensitivity analyses: risk of publication bias for blood pressure outcomes

Appendix F. Forrest plots: effect of sleep interventions on heart rate

Appendix G. Forrest plots: effect of sleep interventions on heart rate variability

Appendix H. Bubble plots: relationship between blood pressure and proportion of males

Appendix I. Sensitivity analysis: forrest plots according to the risk of bias categories

Appendix J. Sensitivity analysis: 'leave one-out-analysis'

Appendix K. Sub-analysis: Forrest plots for blood pressure according to baseline sleep-problem phenotype.

Appendix L. GRADE assessment summary of findings

Appendix A: Database search strategy

1. MEDLINE search strategy

Ovid MEDLINE(R) ALL <1946 to December 18, 2024>

1 Chronotype.ab. or Chronotype.ti.
2 (Circadian adj1 (rhythm or misalignment or clock*)).ab. or (Circadian adj1 (rhythm or misalignment or
clock*)).ti.
3 (Morning* adj2 Evening*).ab. or (Morning* adj2 Evening*).ti.
4 "Biological Clock*".ab. or "Biological Clock*".ti.
5 "Social jetlag".ab. or "Social jetlag".ti.
6 (Sleep adj1 (duration or disorder* or "Initiation and Maintenance Disorder" or stage* or "Apn?ea
Syndrome*" or "Apn?ea Obstructive" or "wake disorder*" or "disordered breathing" or DYSFUNTION or
Problem*)).ab. or (Sleep adj1 (duration or disorder* or "Initiation and Maintenance Disorder" or stage* or
"Apn?ea Syndrome*" or "Apn?ea Obstructive" or "wake disorder*" or "disordered breathing" or
DYSFUNTION or Problem*)).ti.
7 (Nap or naps or napping).ab. or (Nap or naps or napping).ti.
8 wakefulness.ab. or wakefulness.ti.
9 "shift work*".ab. or "shift work*".ti.
10 insomnia.ab. or insomnia.ti.
11 hyposomni*.ab. or hyposomni*.ti.
12 sleepless.ab. or sleepless.ti.
13 (early adj1 (awake* or wake or wakes or waking)).ab. or (early adj1 (awake* or wake or wakes or
waking)).ti.
14 ((difficult* or disturb* or inabilit* or unable* or problem* or reduced) adj3 (asleep or sleep*)).ab. or
((difficult* or disturb* or inabilit* or unable* or problem* or reduced) adj3 (asleep or sleep*)).ti.
15 exp sleep deprivation/ or exp sleep disorders, circadian rhythm/ or exp sleep disorders, intrinsic/
16 exp Circadian Rhythm/
17 exp Sleep Stages/
18 1 or 2 or 3 or 4 or 5 or 6 or 7 or 8 or 9 or 10 or 11 or 12 or 13 or 14 or 15 or 16 or 17
19 "cognitive behavio?ral therap*".ab. or "cognitive behavio?ral therap*".ti.
20 "cognitive therap*".ab. or "cognitive therap*".ti.
21 (Sleep adj1 (hygiene or education or program* or advice or extension)).ab. or (Sleep adj1 (hygiene or
education or program* or advice or extension)).ti.
22 "bright light".ab. or "bright light".ti.
23 "stimulus control".ab. or "stimulus control".ti.
24 relaxation.ab. or relaxation.ti.
25 meditation.ab. or meditation.ti.
26 mindfulness.ab. or mindfulness.ti.
27 non-pharmacological.ab. or non-pharmacological.ti.
28 exp Cognitive Behavioral Therapy/
29 Sleep Hygiene/
30 meditation/ or relaxation therapy/
31 Mindfulness/

32 19 or 20 or 21 or 22 or 23 or 24 or 25 or 26 or 27 or 28 or 29 or 30 or 31
33 "blood pressure*".ab. or "blood pressure*".ti.
34 Blood Pressure/
35 Hypertensi*.ab. or Hypertensi*.ti.
36 Pre-hypertensi*.ab. or Pre-hypertensi*.ti.
37 exp Hypertension/
38 ((heart or pulse) adj1 rate*).ab. or ((heart or pulse) adj1 rate*).ti.
39 ("cardiac Vagal" adj1 (control or tone)).ab. or ("cardiac Vagal" adj1 (control or tone)).ti.
40 "Autonomic Nervous System".ab. or "Autonomic Nervous System".ti.
41 "Cardio-Vascular Reactivity".ab. or "Cardio-Vascular Reactivity".ti.
42 Heart Rate/
43 33 or 34 or 35 or 36 or 37 or 38 or 39 or 40 or 41 or 42
44 18 and 32 and 43

2. Embase <1974 to 2024 December 18>

1 Chronotype.ab. or Chronotype.ti.
2 (Circadian adj1 (rhythm or misalignment or clock*)).ab. or (Circadian adj1 (rhythm or misalignment or clock*)).ti.
3 (Morning* adj2 Evening*).ab. or (Morning* adj2 Evening*).ti.
4 "Biological Clock*".ab. or "Biological Clock*".ti.
5 "Social jetlag".ab. or "Social jetlag".ti.
6 (Sleep adj1 (duration or disorder* or "Initiation and Maintenance Disorder" or stage* or "Apn?ea Syndrome*" or "Apn?ea Obstructive" or "wake disorder*" or "disordered breathing" or DYSFUNTION or Problem*)).ab. or (Sleep adj1 (duration or disorder* or "Initiation and Maintenance Disorder" or stage* or "Apn?ea Syndrome*" or "Apn?ea Obstructive" or "wake disorder*" or "disordered breathing" or DYSFUNTION or Problem*)).ti.
7 (Nap or naps or napping).ab. or (Nap or naps or napping).ti.
8 wakefulness.ab. or wakefulness.ti.
9 "shift work*".ab. or "shift work*".ti.
10 insomnia.ab. or insomnia.ti.
11 hyposomni*.ab. or hyposomni*.ti.
12 sleepless.ab. or sleepless.ti.
13 (early adj1 (awake* or wake or wakes or waking)).ab. or (early adj1 (awake* or wake or wakes or waking)).ti.
14 ((difficult* or disturb* or inabilit* or unable* or problem* or reduced) adj3 (asleep or sleep*)).ab. or ((difficult* or disturb* or inabilit* or unable* or problem* or reduced) adj3 (asleep or sleep*)).ti.
15 exp sleep deprivation/ or exp sleep disorders, circadian rhythm/ or exp sleep disorders, intrinsic/
16 exp Circadian Rhythm/
17 exp Sleep Stages/
18 1 or 2 or 3 or 4 or 5 or 6 or 7 or 8 or 9 or 10 or 11 or 12 or 13 or 14 or 15 or 16 or 17
19 "cognitive behavio?ral therap*".ab. or "cognitive behavio?ral therap*".ti.
20 "cognitive therap*".ab. or "cognitive therap*".ti.
21 (Sleep adj1 (hygiene or education or program* or advice or extension)).ab. or (Sleep adj1 (hygiene or education or program* or advice or extension)).ti.
22 "bright light".ab. or "bright light".ti.
23 "stimulus control".ab. or "stimulus control".ti.
24 relaxation.ab. or relaxation.ti.
25 meditation.ab. or meditation.ti.
26 mindfulness.ab. or mindfulness.ti.
27 non-pharmacological.ab. or non-pharmacological.ti.
28 exp Cognitive Behavioral Therapy/

29 Sleep Hygiene/
30 meditation/ or relaxation therapy/
31 Mindfulness/
32 19 or 20 or 21 or 22 or 23 or 24 or 25 or 26 or 27 or 28 or 29 or 30 or 31
33 "blood pressure*".ab. or "blood pressure*".ti.
34 Blood Pressure/
35 Hypertensi*.ab. or Hypertensi*.ti.
36 Pre-hypertensi*.ab. or Pre-hypertensi*.ti.
37 exp Hypertension/
38 ((heart or pulse) adj1 rate*).ab. or ((heart or pulse) adj1 rate*).ti.
39 ("cardiac Vagal" adj1 (control or tone)).ab. or ("cardiac Vagal" adj1 (control or tone)).ti.
842 40 "Autonomic Nervous System".ab. or "Autonomic Nervous System".ti.
41 "Cardio-Vascular Reactivity".ab. or "Cardio-Vascular Reactivity".ti.
42 Heart Rate/
43 33 or 34 or 35 or 36 or 37 or 38 or 39 or 40 or 41 or 42
44 18 and 32 and 43

3. Cochrane Library

Date Run: 19/12/2024
#1 MeSH descriptor: [Sleep Stages] explode all trees
#2 MeSH descriptor: [Circadian Rhythm] explode all trees
#3 MeSH descriptor: [Sleep Deprivation] explode all trees
#4 MeSH descriptor: [Sleep Wake Disorders] explode all trees
#5 Chronotype OR (Circadian ADJ1 (rhythm or misalignment or clock*)) OR (Morning* ADJ2 Evening*) OR (Biological NEXT Clock*) OR (SOCIAL NEXT JETLAG*) OR (Sleep ADJ1 (duration or disorder* or "Initiation and Maintenance Disorder" or stage* or Apnea NEXT Syndrome* or "Apnea Obstructive" or apnoea NEXT syndrome* OR "apnoea obstructive" or (wake NEXT disorder*) or "disordered breathing" or DYSFUNTION or Problem*)) OR Nap or naps or napping OR wakefulness OR (SHIFT NEXT WORK*) OR insomnia OR hyposomni* OR sleepless OR (early ADJ1 (awake* or wake or wakes or waking)) OR ((difficult* or disturb* or inabilit* or unable* or problem* or reduced) ADJ3 (asleep or sleep*))
#6 #1 OR #2 OR #3 OR #4 OR #5
#7 ("cognitive behavioral" NEXT therap*) OR ("cognitive behavioural" NEXT therap*) or (Cognitive NEXT therap*) OR (Sleep ADJ1 (hygiene or education or program* or advice or extension)) OR "bright light" OR "stimulus control" OR relaxation OR meditation OR mindfulness OR non-pharmacological
#8 MeSH descriptor: [Cognitive Behavioral Therapy] explode all trees
#9 MeSH descriptor: [Sleep Hygiene] this term only
#10 MeSH descriptor: [Meditation] this term only
#11 MeSH descriptor: [Mindfulness] this term only
#12 MeSH descriptor: [Relaxation Therapy] this term only
#13 #7 or #8 or #9 or #10 or #11 or #12
#14 MeSH descriptor: [Blood Pressure] explode all trees
#15 MeSH descriptor: [Heart Rate] explode all trees
#16 MeSH descriptor: [Hypertension] this term only
#17 (blood NEXT pressure*) OR (heart NEXT rate*) OR Hypertensi* OR Pre-hypertensi* OR (pulse NEXT rate*) OR ("cardiac Vagal" adj1 (control or tone)) OR "Autonomic Nervous System" OR "Cardio-Vascular Reactivity"
#18 #14 or #15 or #16 or #17
#19 #6 AND #13 AND #18
230 TRIALS

4. CINAHL

S18 AND S32 AND S42
S33 OR S34 OR S35 OR S36 OR S37 OR S38 OR S39 OR S40 OR S41
(MH "Heart Rate")
(MH "Blood Pressure") OR (MH "Hypertension")
TI "Cardio-Vascular Reactivity" OR AB "Cardio-Vascular Reactivity"
TI "Autonomic Nervous System" OR AB "Autonomic Nervous System"
TI (("cardiac Vagal" N1 (control or tone))) OR AB (("cardiac Vagal" N1 (control or tone)))
TI (((heart or pulse) N1 rate*)) OR AB (((heart or pulse) N1 rate*))
TI Pre-hypertensi* OR AB Pre-hypertensi*
AB Hypertensi* OR TI Hypertensi*
TI "blood pressure*" OR AB "blood pressure*"
S19 OR S20 OR S21 OR S22 OR S23 OR S24 OR S25 OR S26 OR S27 OR S28 OR S29 OR S30 OR S31
(MH "Mindfulness+")
(MH "Relaxation") OR (MH "Relaxation Techniques+")
(MH "Sleep Hygiene+")
(MH "Cognitive Therapy+")
TI non-pharmacological OR AB non- pharmacological
TI mindfulness OR AB mindfulness
TI meditation OR AB meditation
TI relaxation OR AB relaxation
TI "stimulus control" OR AB "stimulus control"
TI "bright light" OR AB "bright light"
TI ((Sleep N1 (hygiene or education or program* or advice or extension))) OR AB ((Sleep N1 (hygiene or education or program* or advice or extension)))
TI "cognitive therap*" OR AB "cognitive therap*"
TI "cognitive behavio#ral therap*" OR AB "cognitive behavio#ral therap*"
S1 OR S2 OR S3 OR S4 OR S5 OR S6 OR S7 OR S8 OR S9 OR S10 OR S11 OR S12 OR S13 OR S14 OR S15 OR S16 OR S17
(MH "Sleep Stages+")
(MH "Circadian Rhythm+")
(MH "Sleep Deprivation") OR (MH "Sleep Disorders, Circadian Rhythm+") OR (MH "Sleep Disorders, In-trinsic+")
TI (((difficult* or disturb* or inabilit* or unable* or problem* or reduced) N3 (asleep or sleep*))) OR AB (((difficult* or disturb* or inabilit* or unable* or problem* or reduced) N3 (asleep or sleep*)))
TI ((early N1 (awake* or wake or wakes or waking))) OR AB ((early N1 (awake* or wake or wakes or wak-ing)))
TI sleepless OR AB sleepless
TI hyposomni* OR AB hyposomni*
TI insomnia OR AB insomnia
TI "shift work*" OR AB "shift work*"
TI wakefulness OR AB wakefulness
TI (Nap or naps or napping) OR AB (Nap or naps or napping)
TI ((Sleep N1 (duration or disorder* or "Initiation and Maintenance Disorder" or stage* or "Apn?ea Syn-drome*" or "Apn?ea Obstructive" or "wake disorder*" or "disordered breathing" or DYSFUNTION or Prob-lem*))) OR AB ((Sleep N1 (duration or disorder* or "Initiation and Maintenance Disorder" or stage* or "Apn?ea Syndrome*" or "Apn?ea Obstructive" or "wake disorder*" or "disordered breathing" or DYS-FUNTION or Problem*)))
TI "Social jetlag" OR AB "Social jetlag"
TI "Biological Clock*" OR AB "Biological Clock*"
TI (Morning* N2 Evening*) OR AB (Morning* N2 Evening*)
TI ((Circadian N1 (rhythm or misalignment or clock*))) OR AB ((Circadian N1 (rhythm or misalignment or clock*)))
TI Chronotype OR AB Chronotype

S18 AND S32 AND S42
S33 OR S34 OR S35 OR S36 OR S37 OR S38 OR S39 OR S40 OR S41
(MH "Heart Rate")
(MH "Blood Pressure") OR (MH "Hypertension")
TI "Cardio-Vascular Reactivity" OR AB "Cardio-Vascular Reactivity"
TI "Autonomic Nervous System" OR AB "Autonomic Nervous System"
TI (("cardiac Vagal" N1 (control or tone))) OR AB (("cardiac Vagal" N1 (control or tone)))
TI (((heart or pulse) N1 rate*)) OR AB (((heart or pulse) N1 rate*))
TI Pre-hypertensi* OR AB Pre-hypertensi*
AB Hypertensi* OR TI Hypertensi*
TI "blood pressure*" OR AB "blood pressure*"
S19 OR S20 OR S21 OR S22 OR S23 OR S24 OR S25 OR S26 OR S27 OR S28 OR S29 OR S30 OR S31
(MH "Mindfulness+")
(MH "Relaxation") OR (MH "Relaxation Techniques+")
(MH "Sleep Hygiene+")
(MH "Cognitive Therapy+")
TI non-pharmacological OR AB non- pharmacological
TI mindfulness OR AB mindfulness
TI meditation OR AB meditation
TI relaxation OR AB relaxation
TI "stimulus control" OR AB "stimulus control"
TI "bright light" OR AB "bright light"
TI ((Sleep N1 (hygiene or education or program* or advice or extension))) OR AB ((Sleep N1 (hygiene or education or program* or advice or extension)))
TI "cognitive therap*" OR AB "cognitive therap*"
TI "cognitive behavio#ral therap*" OR AB "cognitive behavio#ral therap*"
S1 OR S2 OR S3 OR S4 OR S5 OR S6 OR S7 OR S8 OR S9 OR S10 OR S11 OR S12 OR S13 OR S14 OR S15 OR S16 OR S17
(MH "Sleep Stages+")
(MH "Circadian Rhythm+")
(MH "Sleep Deprivation") OR (MH "Sleep Disorders, Circadian Rhythm+") OR (MH "Sleep Disorders, In-trinsic+")
TI (((difficult* or disturb* or inabilit* or unable* or problem* or reduced) N3 (asleep or sleep*))) OR AB (((difficult* or disturb* or inabilit* or unable* or problem* or reduced) N3 (asleep or sleep*)))
TI ((early N1 (awake* or wake or wakes or waking))) OR AB ((early N1 (awake* or wake or wakes or wak-ing)))
TI sleepless OR AB sleepless
TI hyposomni* OR AB hyposomni*
TI insomnia OR AB insomnia
TI "shift work*" OR AB "shift work*"
TI wakefulness OR AB wakefulness
TI (Nap or naps or napping) OR AB (Nap or naps or napping)
TI ((Sleep N1 (duration or disorder* or "Initiation and Maintenance Disorder" or stage* or "Apn?ea Syn-drome*" or "Apn?ea Obstructive" or "wake disorder*" or "disordered breathing" or DYSFUNTION or Prob-lem*))) OR AB ((Sleep N1 (duration or disorder* or "Initiation and Maintenance Disorder" or stage* or "Apn?ea Syndrome*" or "Apn?ea Obstructive" or "wake disorder*" or "disordered breathing" or DYS-FUNTION or Problem*)))
TI "Social jetlag" OR AB "Social jetlag"
TI "Biological Clock*" OR AB "Biological Clock*"
TI (Morning* N2 Evening*) OR AB (Morning* N2 Evening*)
TI ((Circadian N1 (rhythm or misalignment or clock*))) OR AB ((Circadian N1 (rhythm or misalignment or clock*)))
TI Chronotype OR AB Chronotype

Appendix B. Risk of bias assessment

A) Traffic-light plot for Randomised trials

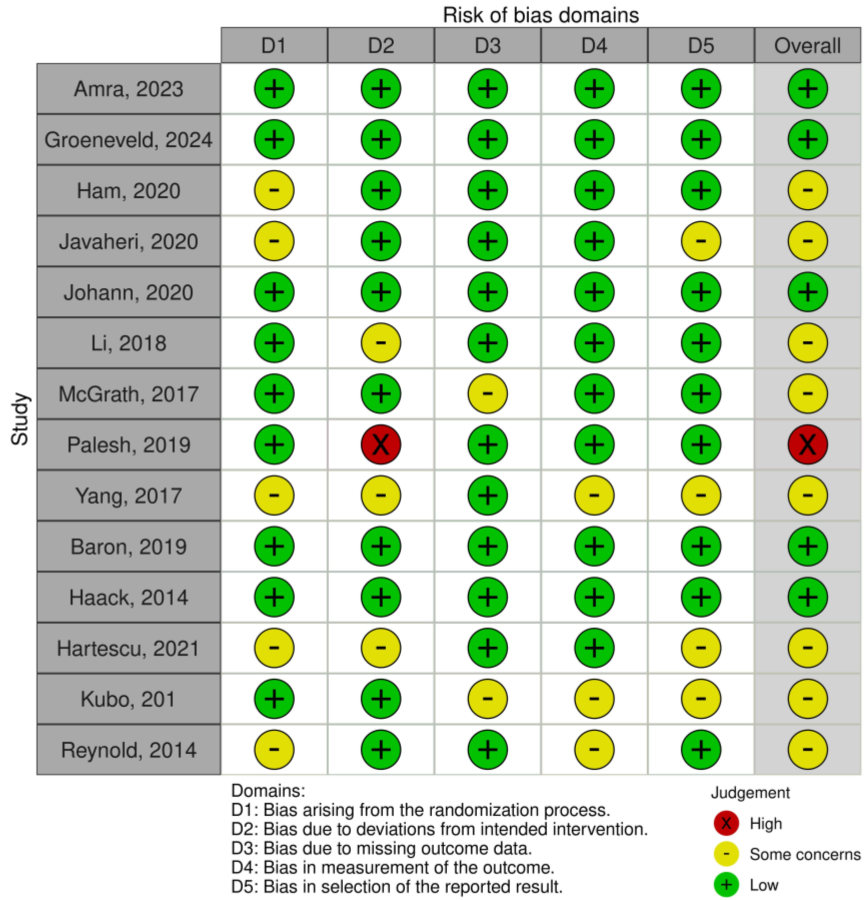

B) Weighted-summary plot for Randomised trials

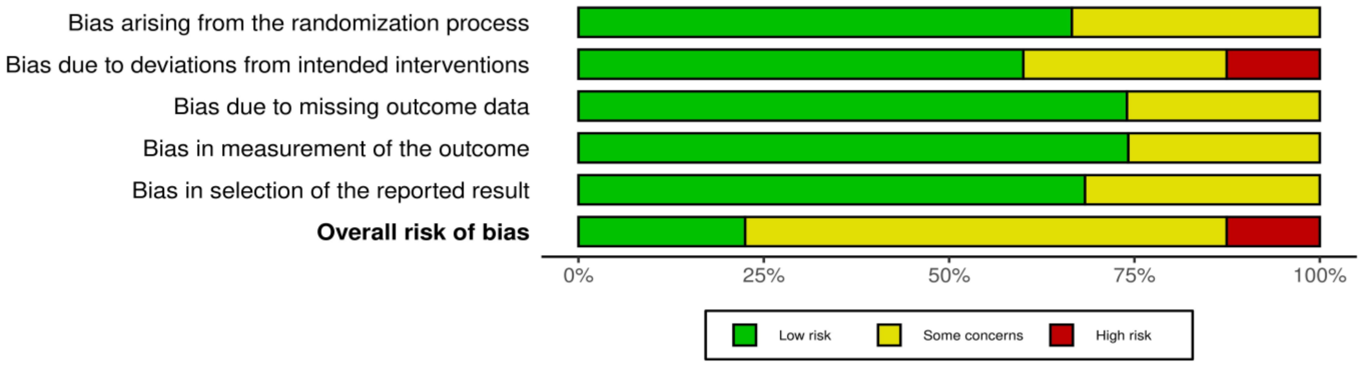

C) Traffic light plot for non-randomised trials ('pre-post' single-arm cross-over trials)

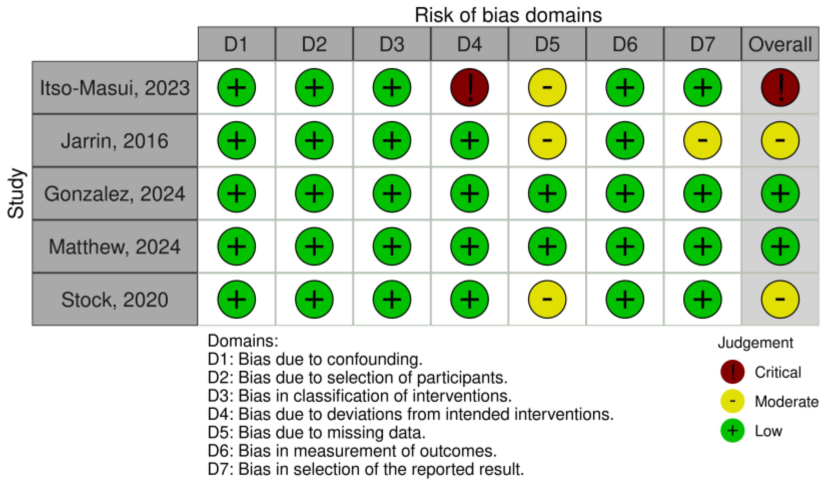

D) Weighted summary plot for non-randomised trials

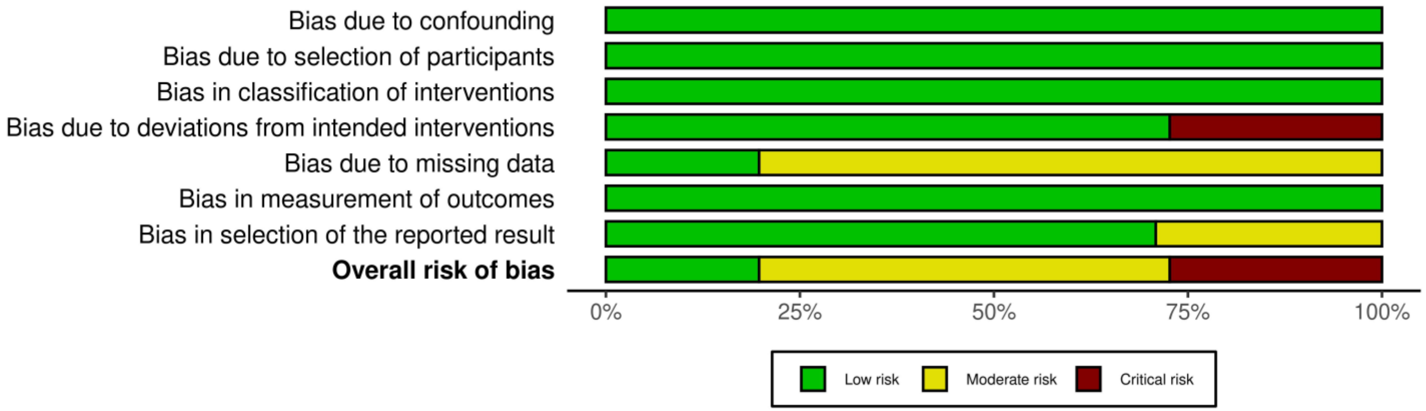

Randomised studies were analysed using the Cochrane risk-of-bias tool for randomised trials version 2 (ROB-2) tool and non-randomised studies using the Risk of Bias In Non-randomized Studies–of Interventions' (ROBINS-I) tool. Plots were produced using Robvis software (available on: https://mcguinlu.shinyapps.io/robvis/).

Appendix C: Forrest plots for the results of the meta-analysis examining the effect of sleep interventions on sleep variables: (a) Insomnia severity index and Pittsburgh Sleep Quality Index global score and (b) Sleep duration and Time in bed.

a) Insomnia severity index and Pittsburgh Sleep Quality Index global score

	Intervention			Control				Mean Difference
Study or Subgroup	Mean	SD	Total	Mean	SD	Total	Weight	IV, Random, 95% CI
**Insomnia severity index**								
Amra 2023	6.7	4.5	31	13.6	6.3	26	11.8%	−6.90 [−9.79, −4.01]
Groeneveld 2024	12.8	5.3	29	13.2	4.8	28	12.2%	−0.40 [−3.02, 2.22]
Ham 2020	9.78	4.09	24	14.39	6.26	20	11.2%	−4.61 [−7.80, −1.42]
Jarrin 2016	8.8	3.5	65	17	4	65	14.2%	−8.20 [−9.49, −6.91]
Javaheri 2020	−6.2	5.3	15	−3.3	5.1	14	10.2%	−2.90 [−6.69, 0.89]
Johann 2020	6.5	3.8	23	13	4.7	23	12.5%	−6.50 [−8.97, −4.03]
McGrath 2017	−4.5	4.4	54	−1.7	4.1	67	13.9%	−2.80 [−4.33, −1.27]
Yang 2021	8.53	3.2	53	10.88	4.35	53	14.0%	−2.35 [−3.80, −0.90]
**Subtotal (95% CI)**			**294**			**296**	**100.0%**	**−4.37 [−6.52, −2.21]**
Heterogeneity: Tau² = 8.08; Chi² = 59.60, df = 7 (P < 0.00001); I² = 88%								
Test for overall effect: Z = 3.97 (P < 0.0001)								
**Pittsburgh Sleep Quality Index**								
Ham 2020	8.3	3.4	24	11.11	2.32	20	11.4%	−2.81 [−4.51, −1.11]
Hartescu 2021	−5.55	2.92	10	−2.56	2.62	8	6.7%	−2.99 [−5.55, −0.43]
Ito-Masui 2023	7.84	2.46	61	9.1	4.1	61	15.9%	−1.26 [−2.46, −0.06]
Li 2018	−1.7	0.9	16	−0.4	0.7	15	22.6%	−1.30 [−1.87, −0.73]
McGrath 2017	−2.5	3.1	54	−1.4	2.6	67	17.6%	−1.10 [−2.13, −0.07]
Reynold 2014	4.75	1.75	8	3.67	1.75	6	10.4%	1.08 [−0.77, 2.93]
Yang 2021	9.53	3.21	53	12.26	3.32	53	15.4%	−2.73 [−3.97, −1.49]
**Subtotal (95% CI)**			**226**			**230**	**100.0%**	**−1.52 [−2.29, −0.75]**
Heterogeneity: Tau² = 0.60; Chi² = 15.88, df = 6 (P = 0.01); I² = 62%								
Test for overall effect: Z = 3.86 (P = 0.0001)								

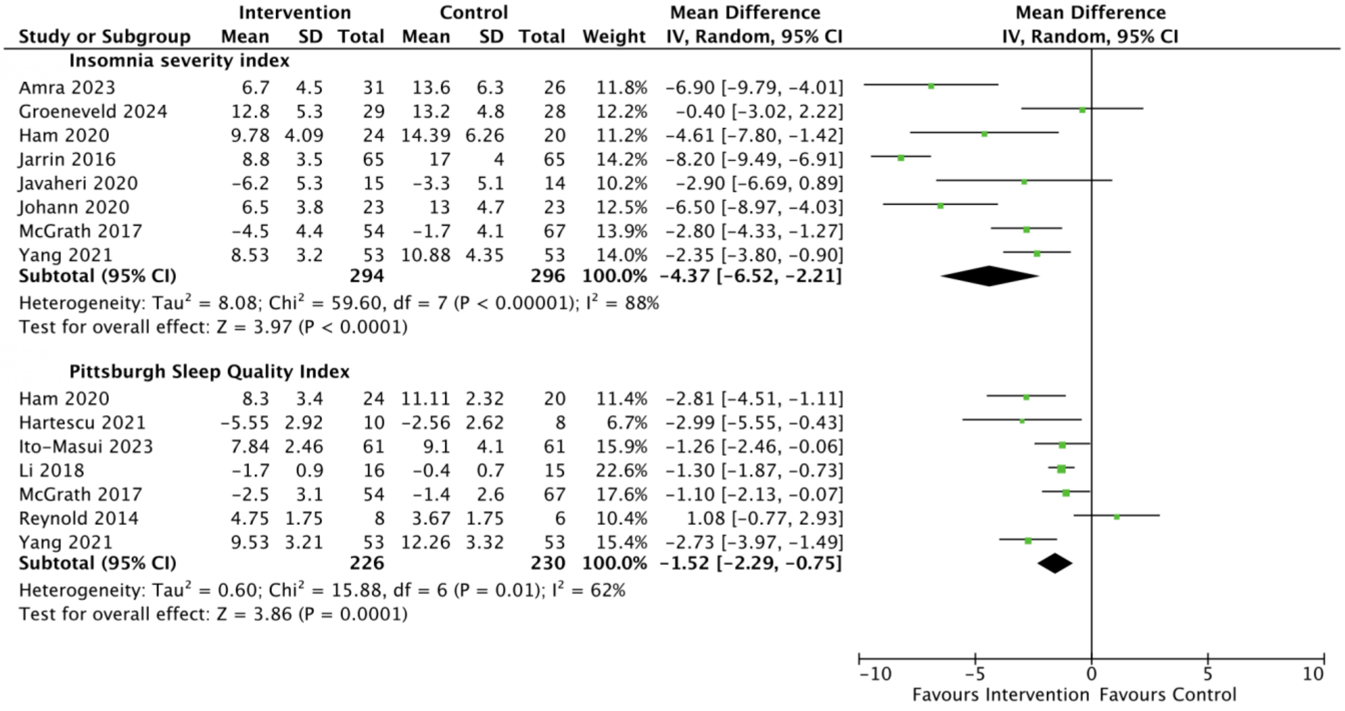

b) Sleep duration and Time-in-bed

	Intervention			Control				Mean Difference
Study or Subgroup	Mean	SD	Total	Mean	SD	Total	Weight	IV, Random, 95% CI
**Sleep duration**								
Gonzalez 2024	549	36	22	405	45	22	14.7%	144.00 [119.92, 168.08]
Groeneveld 2024	414	102	29	444	72	28	11.6%	−30.00 [−75.71, 15.71]
Haack 2013	35	9	13	4	9	9	16.1%	31.00 [23.35, 38.65]
Hartescu 2021	78.48	15.45	10	6.28	15.46	8	15.7%	72.20 [57.83, 86.57]
Ito-Masui 2023	363.6	78	61	332.4	81.6	61	14.1%	31.20 [2.87, 59.53]
Matthew 2023	456	51	12	415.8	51	12	12.3%	40.20 [−0.61, 81.01]
Stock 2020	497	45	53	456.3	38.6	53	15.5%	40.70 [24.74, 56.66]
**Subtotal (95% CI)**			**200**			**193**	**100.0%**	**49.59 [20.49, 78.69]**
Heterogeneity: Tau² = 1354.16; Chi² = 103.13, df = 6 (P < 0.00001); I² = 94%								
Test for overall effect: Z = 3.34 (P = 0.0008)								
**Time-in-bed**								
Baron 2019	453	30.6	11	417	105	5	16.8%	36.00 [−57.79, 129.79]
Gonzalez 2024	633	41	22	468	52	22	21.3%	165.00 [137.33, 192.67]
Groeneveld 2024	498	72	29	522	60	28	21.0%	−24.00 [−58.36, 10.36]
Hartescu 2021	100.01	40.59	10	3.61	41.02	8	20.8%	96.40 [58.44, 134.36]
Reynold 2014	603	58	8	456	34	5	20.1%	147.00 [96.97, 197.03]
**Subtotal (95% CI)**			**80**			**68**	**100.0%**	**85.75 [5.41, 166.09]**
Heterogeneity: Tau² = 7699.56; Chi² = 76.30, df = 4 (P < 0.00001); I² = 95%								
Test for overall effect: Z = 2.09 (P = 0.04)								

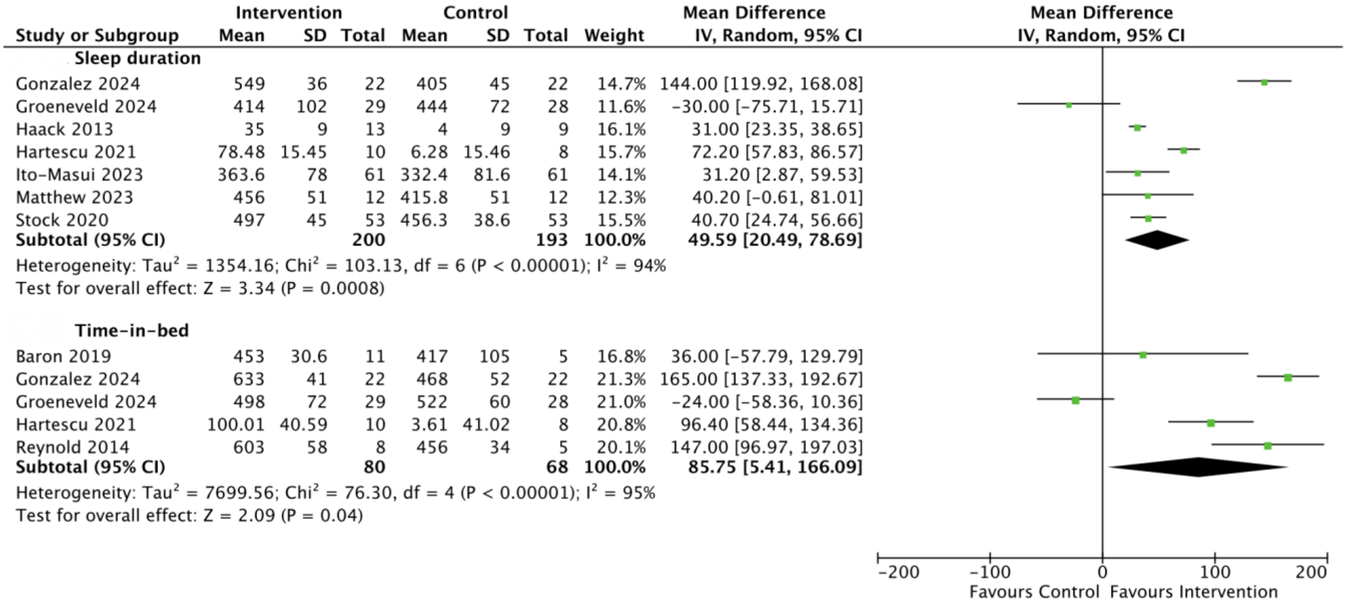

Key: CI= confidence interval, I^2= heterogeneity test, IV = inverse variance, SD = standard deviation. Sleep duration and time-in-bed are measured in minutes.

Appendix D. Forrest plots for the results of the meta-analysis assessing the effect of sleep interventions in non-RCTs only, on levels of systolic and diastolic blood pressure

Study or Subgroup	Intervention Mean	Intervention SD	Intervention Total	Control Mean	Control SD	Control Total	Weight	Mean Difference IV, Random, 95% CI
**SBP**								
Gonzalez 2024	123	16	22	126	16	22	24.2%	−3.00 [−12.46, 6.46]
Matthew 2023	109.18	10.87	11	116.18	12.45	11	22.7%	−7.00 [−16.77, 2.77]
Stock 2020	111.2	14.1	53	117.1	19.1	53	53.0%	−5.90 [−12.29, 0.49]
**Subtotal (95% CI)**			**86**			**86**	**100.0%**	**−5.45 [−10.10, −0.79]**
Heterogeneity: $Tau^2 = 0.00$; $Chi^2 = 0.37$, df = 2 (P = 0.83); $I^2 = 0\%$								
Test for overall effect: Z = 2.29 (P = 0.02)								
**DBP**								
Gonzalez 2024	72	7	22	73	7	22	40.8%	−1.00 [−5.14, 3.14]
Matthew 2023	62.09	6.27	11	66.36	8.05	11	19.2%	−4.27 [−10.30, 1.76]
Stock 2020	68.8	10.5	53	70.9	11.4	53	40.1%	−2.10 [−6.27, 2.07]
**Subtotal (95% CI)**			**86**			**86**	**100.0%**	**−2.07 [−4.71, 0.57]**
Heterogeneity: $Tau^2 = 0.00$; $Chi^2 = 0.77$, df = 2 (P = 0.68); $I^2 = 0\%$								
Test for overall effect: Z = 1.53 (P = 0.12)								

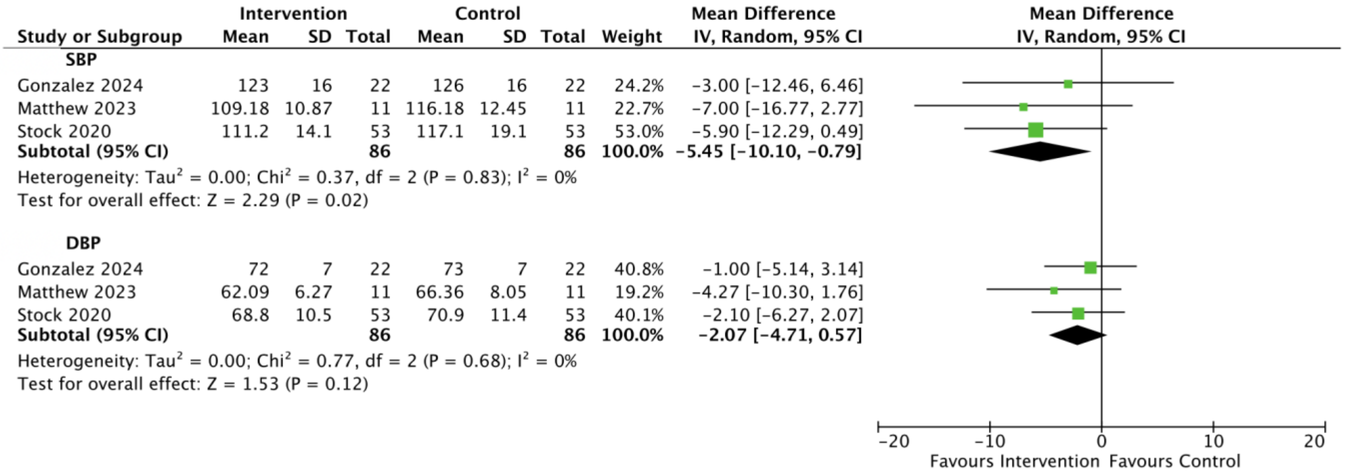

Key: CI = confidence interval, DBP = diastolic blood pressure, I^2= heterogeneity test, IV = inverse variance, SBP = systolic blood pressure SD = standard deviation. All blood pressure variables are measured in mmHg.

Appendix E. Risk of publication bias

(1) Funnel plots before and after the removal of 11 smaller studies

(a) Systolic blood pressure (SBP)
(i) All studies in the outcome (n=15)

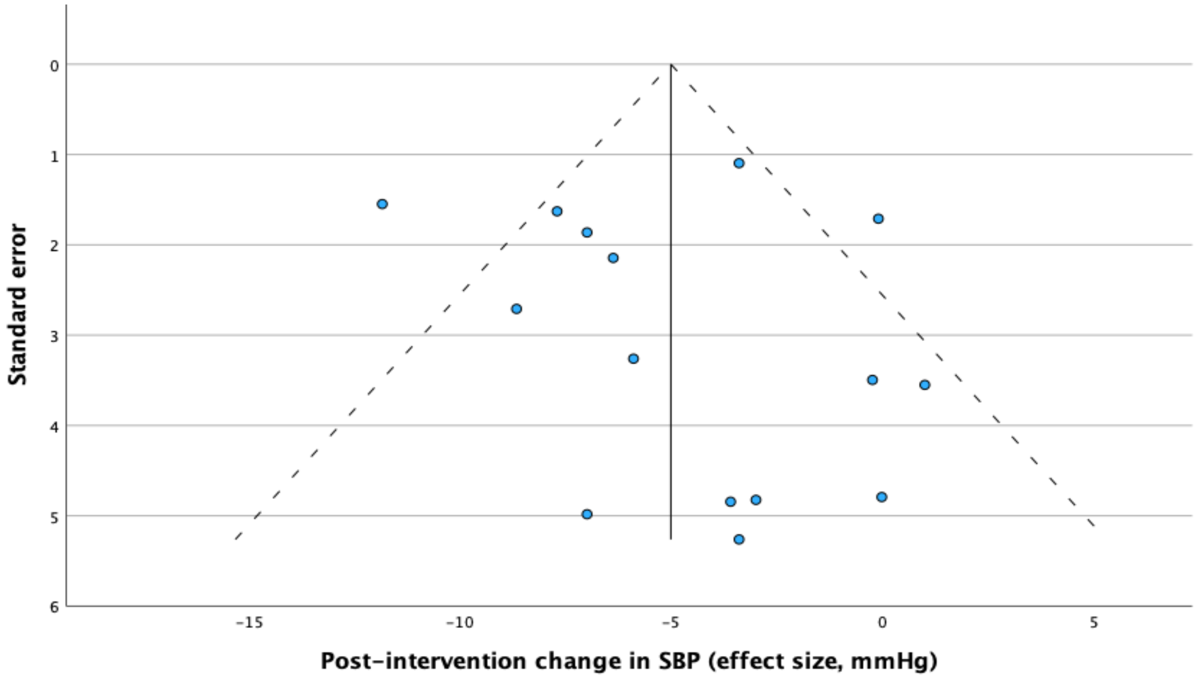

(ii) After removal of 11 smaller studies

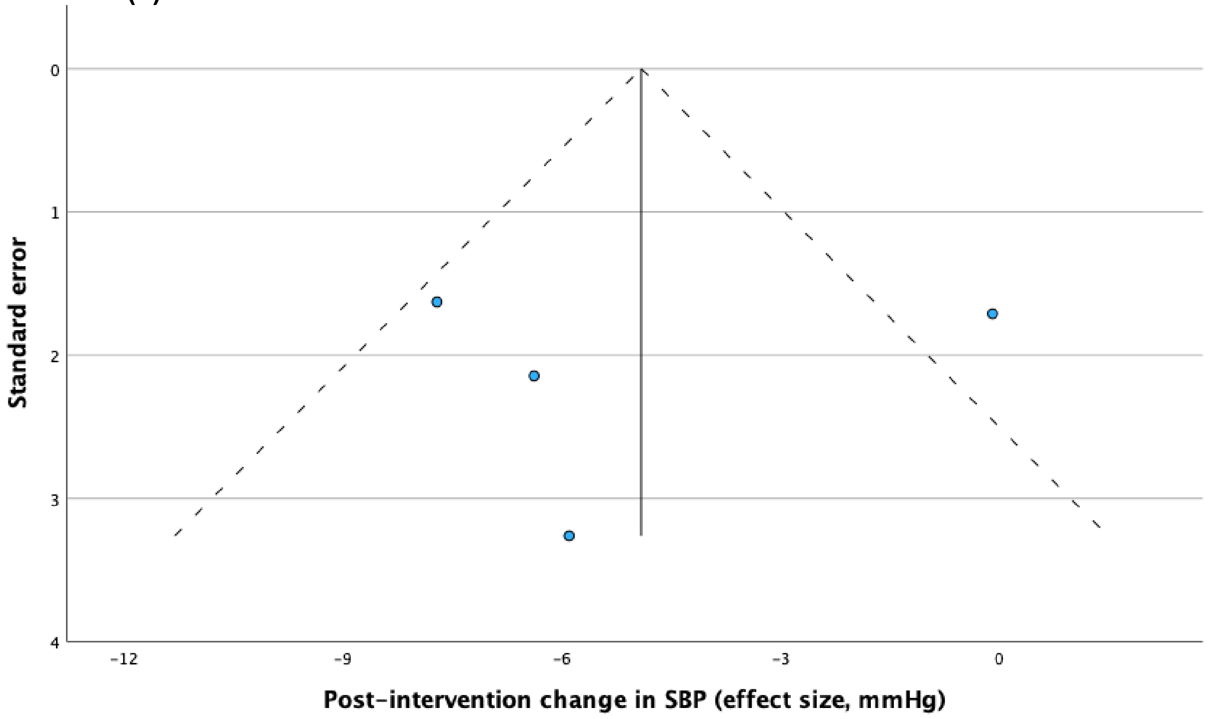

(b) Diastolic blood pressure (DBP)
(i) For all studies (n=15)

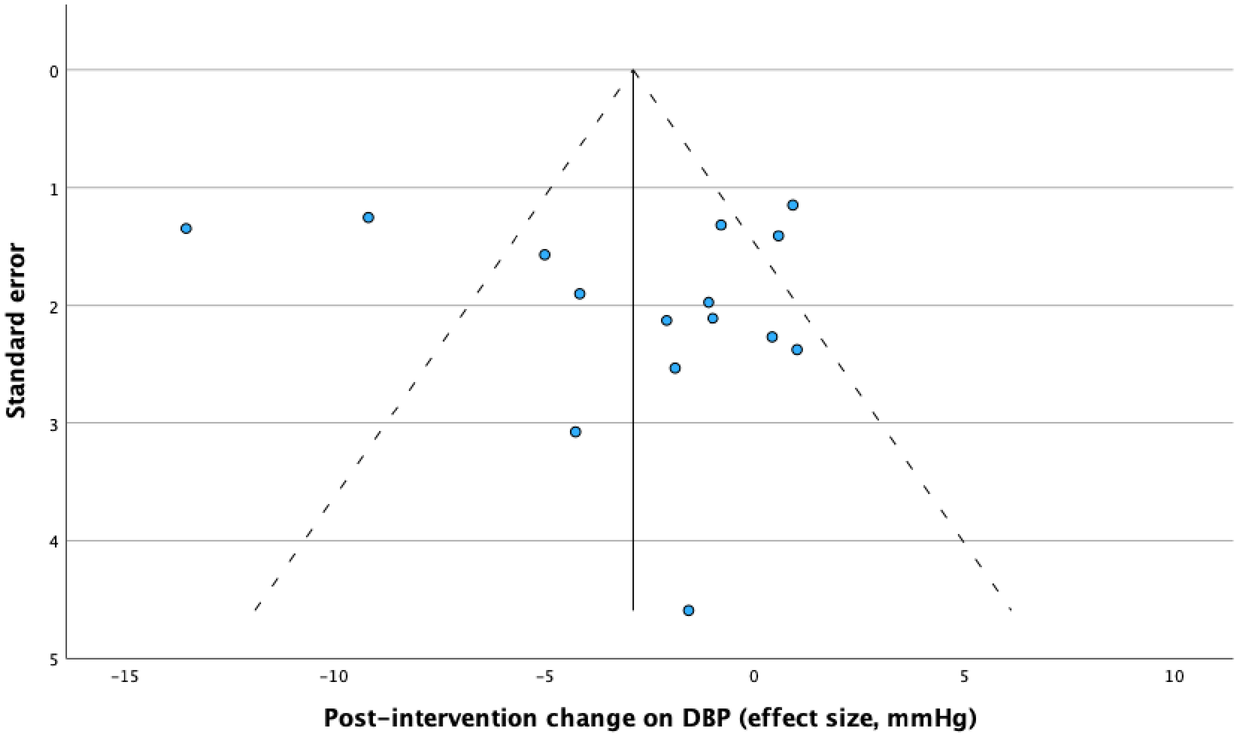

(ii) After removal of 11 smaller studies

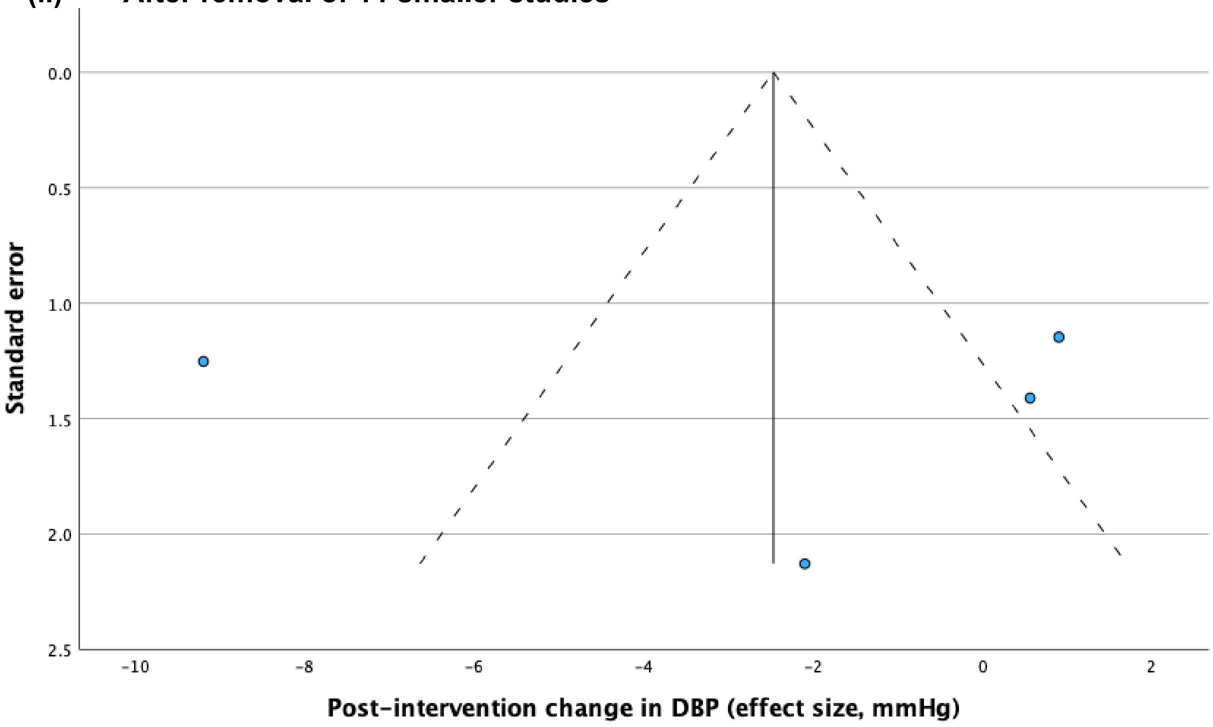

Each dot represents an individual study, the continuous vertical line represents the overall effect size and the dotted line represents 95% confidence intervals.11 smaller studies with less than 60 participants were removed (reference: Teare MD et al. Trials. 2014; 15:264. doi:

10.1186/1745-6215-15-264). The remaining four studies (n=517 participants) were re-analysed. Overall, no true asymmetry is demonstrated suggesting a lower risk of publication bias.

2) Forrest plots for the results of meta-analyses after removing smaller studies (n=11) from the main outcome analyses for systolic and diastolic blood pressure.

	Intervention			Control				Mean Difference
Study or Subgroup	Mean	SD	Total	Mean	SD	Total	Weight	IV, Random, 95% CI
SBP								
Liu 2018	−12.64	10.52	102	−4.93	11.34	82	28.6%	−7.71 [−10.90, −4.52]
McGrath 2017	−0.9	9.4	54	−0.8	9.3	67	28.0%	−0.10 [−3.45, 3.25]
Stock 2020	111.2	14.1	53	117.1	19.1	53	18.3%	−5.90 [−12.29, 0.49]
Yang 2021	128.76	10.79	53	135.14	11.29	53	25.1%	−6.38 [−10.58, −2.18]
**Subtotal (95% CI)**			**262**			**255**	**100.0%**	**−4.91 [−8.86, −0.96]**
Heterogeneity: $Tau^2 = 11.59$; $Chi^2 = 11.39$, df = 3 (P = 0.010); $I^2 = 74\%$								
Test for overall effect: Z = 2.44 (P = 0.01)								
DBP								
Liu 2018	−5.97	7.67	102	3.23	9.02	82	25.7%	−9.20 [−11.66, −6.74]
McGrath 2017	−0.6	6	54	−1.5	6.6	67	25.9%	0.90 [−1.35, 3.15]
Stock 2020	68.8	10.5	53	70.9	11.4	53	23.2%	−2.10 [−6.27, 2.07]
Yang 2021	78.86	7	53	78.3	7.52	53	25.3%	0.56 [−2.21, 3.33]
**Subtotal (95% CI)**			**262**			**255**	**100.0%**	**−2.47 [−7.67, 2.73]**
Heterogeneity: $Tau^2 = 25.84$; $Chi^2 = 42.13$, df = 3 (P < 0.00001); $I^2 = 93\%$								
Test for overall effect: Z = 0.93 (P = 0.35)								

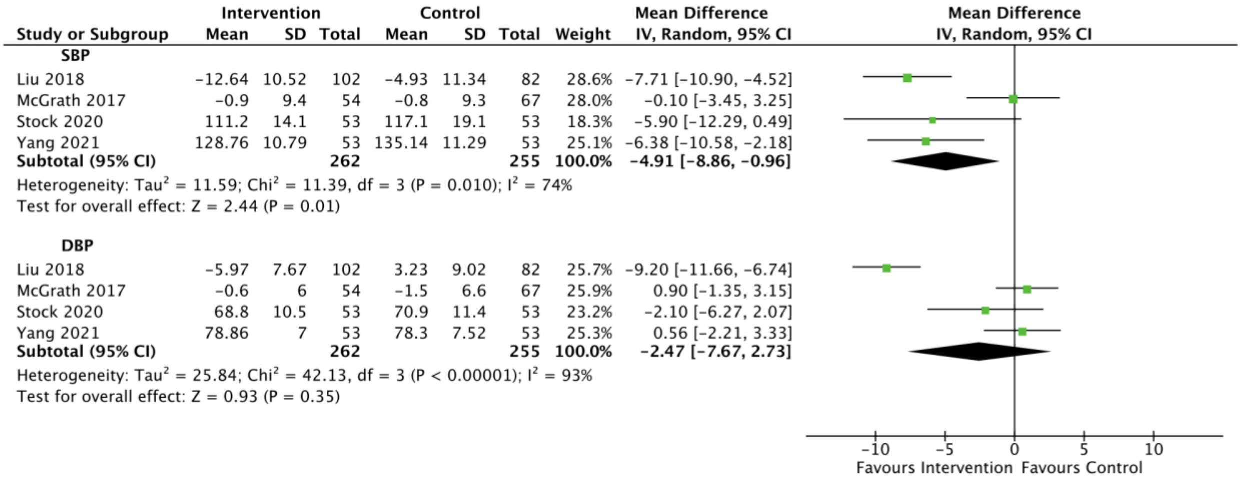

11 smaller studies with less than 60 participants were removed from the total of 15 studies. The remaining four studies (n=517 participants) demonstrated a similar significant systolic blood pressure reduction to those results of the main outcome analysis of the 15 studies, but diastolic blood pressure was no longer significant. Key: CI = confidence interval, DBP = diastolic blood pressure, I^2= heterogeneity test, IV = inverse variance, SBP = systolic blood pressure SD = standard deviation. All blood pressure variables are measured in mmHg.

Appendix F. Forrest plots for the results of the meta-analysis assessing the effect of sleep interventions on heart rate

Study or Subgroup	Intervention Mean	Intervention SD	Intervention Total	Control Mean	Control SD	Control Total	Weight	Mean Difference IV, Random, 95% CI
**All interventions**								
Haack 2013	−2	2	13	−1	1	9	27.8%	−1.00 [−2.27, 0.27]
Ito-Masui 2023	74.79	6.62	61	74.9	7.75	61	19.7%	−0.11 [−2.67, 2.45]
Johann 2020	77	7	23	74	9	23	10.3%	3.00 [−1.66, 7.66]
Matthew 2023	70.73	8.86	11	73.91	8.67	11	5.2%	−3.18 [−10.51, 4.15]
Palesh 2019	0.18	9.71	34	8.26	12.89	37	8.7%	−8.08 [−13.36, −2.80]
Reynold 2014	72.58	12.75	8	81.63	7.09	6	2.7%	−9.05 [−19.55, 1.45]
Stock 2020	77.8	13.5	53	80	12	53	9.8%	−2.20 [−7.06, 2.66]
Yang 2021	77.22	9.68	53	77.46	7.23	53	15.9%	−0.24 [−3.49, 3.01]
**Subtotal (95% CI)**			**256**			**253**	**100.0%**	**−1.36 [−3.18, 0.46]**
Heterogeneity: $Tau^2 = 2.68$; $Chi^2 = 13.24$, df = 7 (P = 0.07); $I^2 = 47\%$								
Test for overall effect: Z = 1.46 (P = 0.14)								
**CBT-I +/− Sleep hygiene**								
Ito-Masui 2023	74.79	6.62	61	74.9	7.75	61	30.6%	−0.11 [−2.67, 2.45]
Johann 2020	77	7	23	74	9	23	22.0%	3.00 [−1.66, 7.66]
Palesh 2019	0.18	9.71	34	8.26	12.89	37	19.8%	−8.08 [−13.36, −2.80]
Yang 2021	77.22	9.68	53	77.46	7.23	53	27.7%	−0.24 [−3.49, 3.01]
**Subtotal (95% CI)**			**171**			**174**	**100.0%**	**−1.04 [−4.49, 2.42]**
Heterogeneity: $Tau^2 = 8.47$; $Chi^2 = 10.18$, df = 3 (P = 0.02); $I^2 = 71\%$								
Test for overall effect: Z = 0.59 (P = 0.56)								
**Sleep extension**								
Haack 2013	−2	2	13	−1	1	9	89.9%	−1.00 [−2.27, 0.27]
Matthew 2023	70.73	8.86	11	73.91	8.67	11	2.7%	−3.18 [−10.51, 4.15]
Reynold 2014	72.58	12.75	8	81.63	7.09	6	1.3%	−9.05 [−19.55, 1.45]
Stock 2020	77.8	13.5	53	80	12	53	6.1%	−2.20 [−7.06, 2.66]
**Subtotal (95% CI)**			**85**			**79**	**100.0%**	**−1.24 [−2.44, −0.04]**
Heterogeneity: $Tau^2 = 0.00$; $Chi^2 = 2.68$, df = 3 (P = 0.44); $I^2 = 0\%$								
Test for overall effect: Z = 2.02 (P = 0.04)								

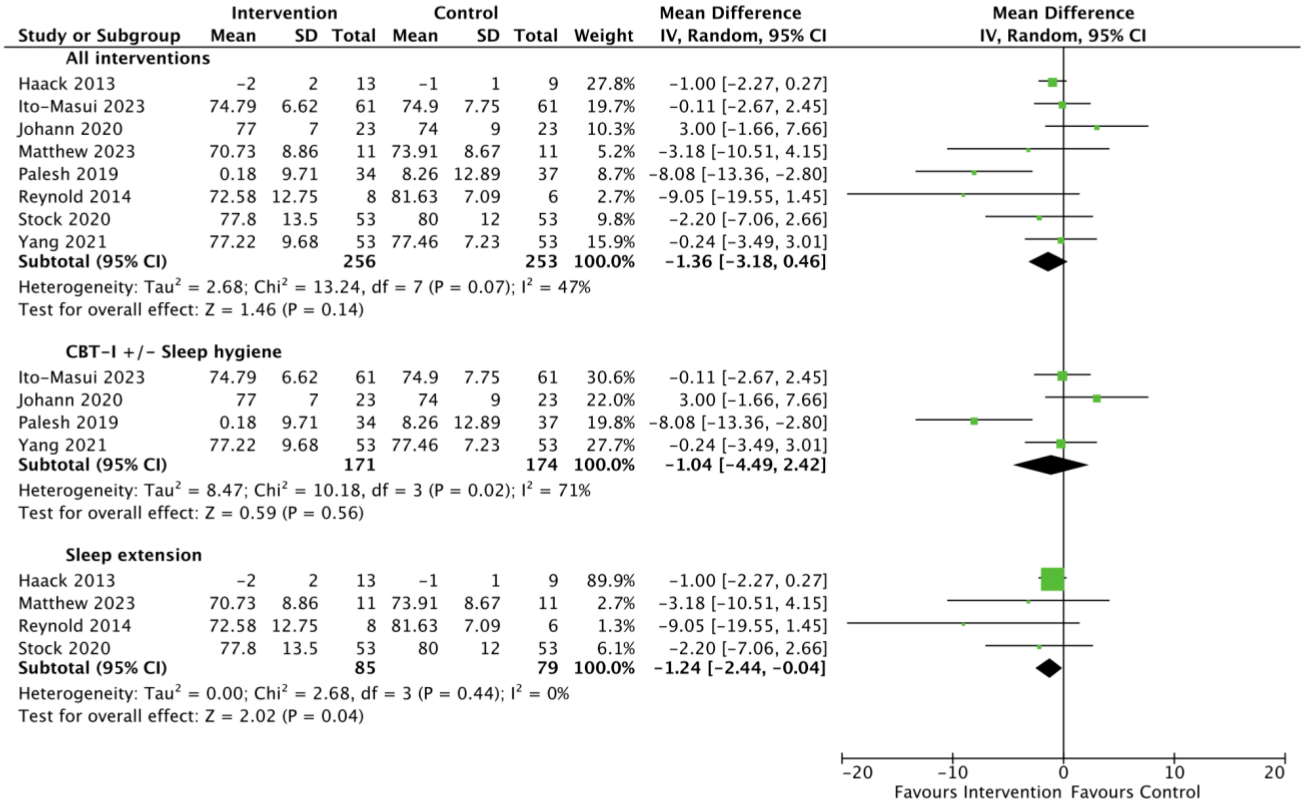

Key: CI = confidence interval, CBT-I = Cognitive Behavioural Therapy for Insomnia, I^2= heterogeneity test, IV = inverse variance, SD = standard deviation. Heart rate is measured in beats per minute. The test for sub-group differences for CBT-I and/ or sleep extension vs sleep extension was not significant for heart rate (p=0.91).

Appendix G. Forrest plots for the results of the meta-analysis assessing the effect of CBT-I on heart rate variability measures

(i) Low-frequency: high-frequency power ratio

Study or Subgroup	Intervention Mean	Intervention SD	Intervention Total	Control Mean	Control SD	Control Total	Weight	Std. Mean Difference IV, Random, 95% CI
Amra 2023	1.1	0.8	31	1.2	0.8	26	18.7%	−0.12 [−0.65, 0.40]
Jarrin 2016	4.7	4.5	65	4.4	4.2	65	43.0%	0.07 [−0.28, 0.41]
Johann 2020	2.1	0.5	23	2.1	0.4	23	15.2%	0.00 [−0.58, 0.58]
Palesh 2019	0.39	1.98	34	1.42	3.4	37	23.1%	−0.36 [−0.83, 0.11]
**Total (95% CI)**			**153**			**151**	**100.0%**	**−0.08 [−0.30, 0.15]**
Heterogeneity: $Tau^2 = 0.00$; $Chi^2 = 2.20$, df = 3 (P = 0.53); $I^2 = 0\%$								
Test for overall effect: Z = 0.67 (P = 0.50)								

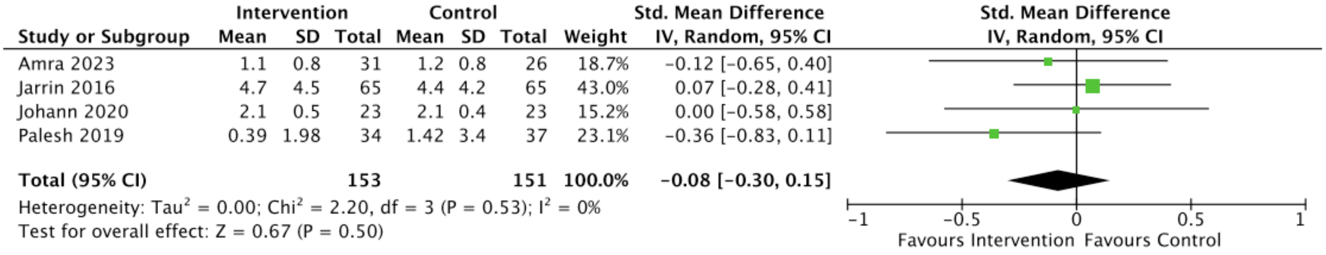

(ii) High Frequency and Standard deviation of normal-to-normal intervals

Study or Subgroup	control Mean	control SD	control Total	Intervention Mean	Intervention SD	Intervention Total	Weight	Std. Mean Difference IV, Random, 95% CI
**HF**								
Amra 2023	45.6	19.8	31	39.8	18.5	26	32.9%	0.30 [−0.23, 0.82]
Jarrin 2016	486.1	682.9	65	396.8	549.9	65	34.6%	0.14 [−0.20, 0.49]
Palesh 2019	0.9	0.83	34	−1.04	1.17	37	32.5%	1.88 [1.31, 2.44]
**Subtotal (95% CI)**			**130**			**128**	**100.0%**	**0.76 [−0.25, 1.77]**
Heterogeneity: $Tau^2 = 0.74$; $Chi^2 = 27.57$, df = 2 (P < 0.00001); $I^2 = 93\%$								
Test for overall effect: Z = 1.47 (P = 0.14)								
**SDNN**								
Johann 2020	48	16	23	51	17	23	48.9%	−0.18 [−0.76, 0.40]
Palesh 2019	0.41	6.6	34	−4.76	4.78	37	51.1%	0.89 [0.40, 1.38]
**Subtotal (95% CI)**			**57**			**60**	**100.0%**	**0.37 [−0.68, 1.42]**
Heterogeneity: $Tau^2 = 0.50$; $Chi^2 = 7.68$, df = 1 (P = 0.006); $I^2 = 87\%$								
Test for overall effect: Z = 0.69 (P = 0.49)								

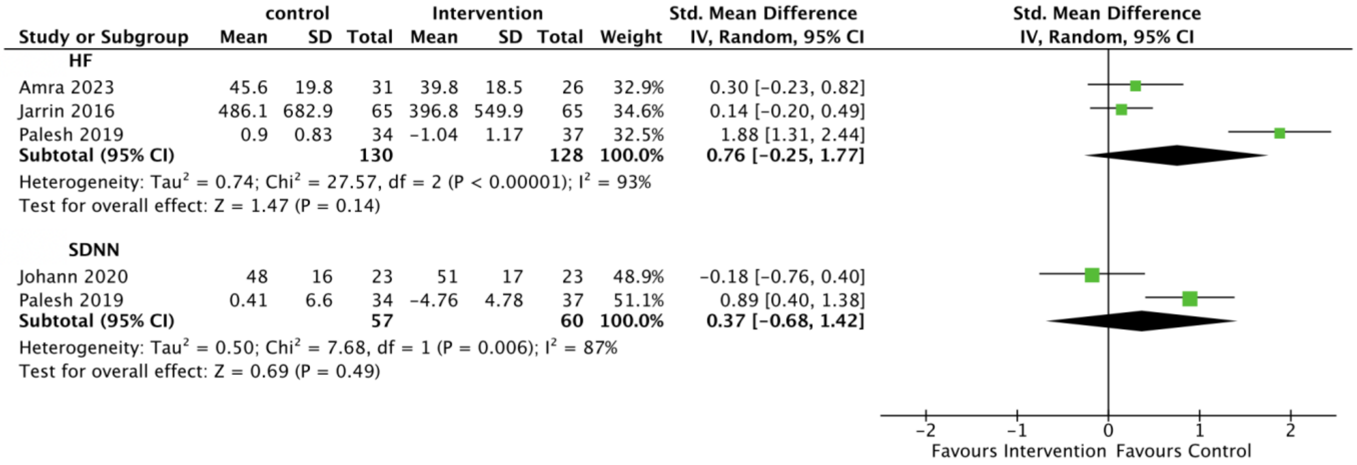

Key: CI = confidence interval, HF = high frequency, I^2= heterogeneity test, IV = inverse variance, SD = standard deviation, SDNN = Standard deviation of normal-to-normal intervals.

Appendix H. Bubble plots for the results of meta-regression models for the association of the portion of males in each study and outcomes of post-intervention change of (a) systolic and (b) diastolic blood pressure

(a) systolic blood pressure

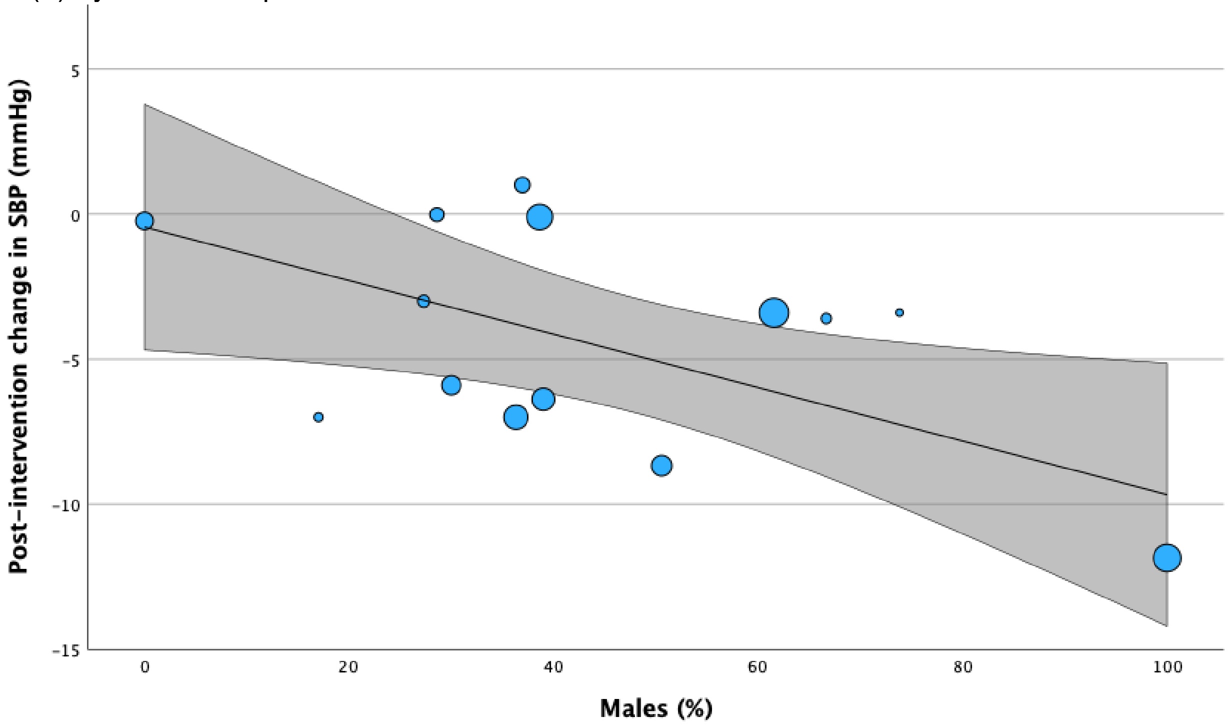

(b) diastolic blood pressure

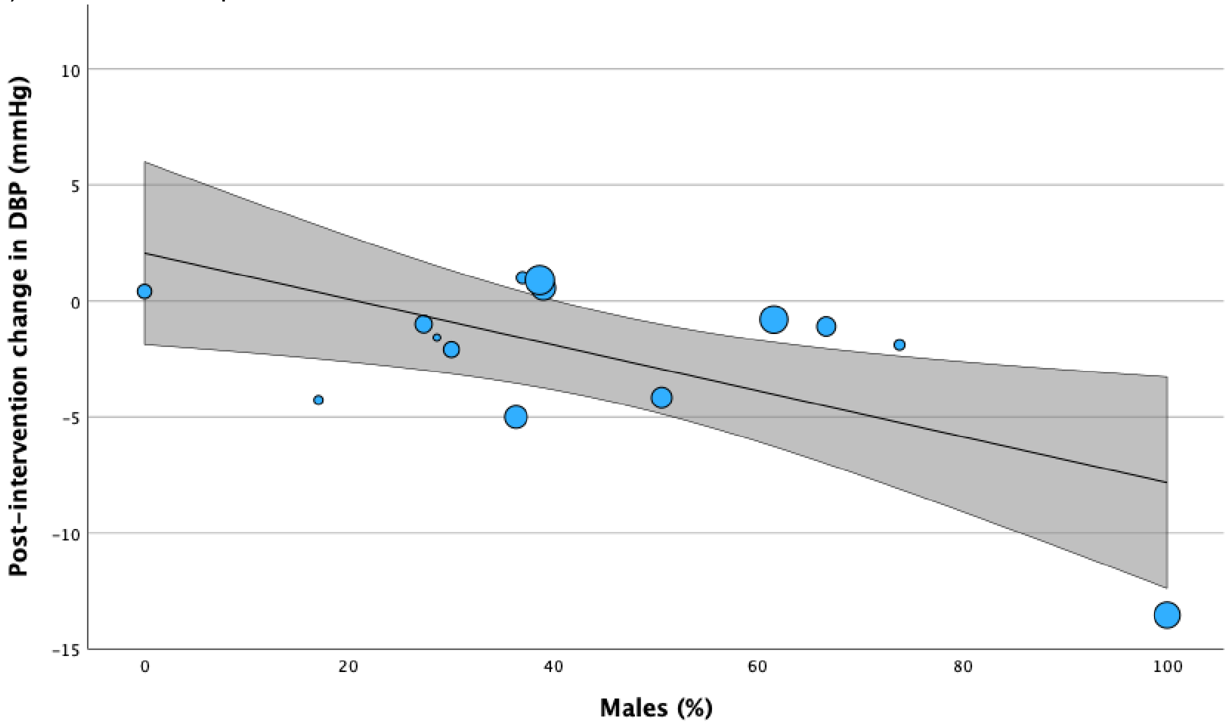

The univariate meta-regression models were formed using random effects function for each

model. Each study, represented by a dot, was assessed for the change in blood pressure (effect size) according to the proportion of males. The larger sized dot represents a stronger weighted study, which in turn have a stronger influence on the meta-regression prediction line. The grey zone represents the 95% confidence intervals. Two single-sex studies with 0 and 100% males are associated with the lowest and highest reductions in blood pressure respectively.

Appendix I. Forrest plots for the results of the meta-analysis according to the risk of bias categories for (a) systolic and (b) diastolic blood pressure.

(a) Systolic blood pressure (mmHg)

	Experimental			Control				Mean Difference
Study or Subgroup	Mean	SD	Total	Mean	SD	Total	Weight	IV, Random, 95% CI
**Low**								
Baron 2019	−8.67	7.63	11	0	3.2	5	22.5%	−8.67 [−13.98, −3.36]
Gonzalez 2024	123	16	22	126	16	22	8.2%	−3.00 [−12.46, 6.46]
Groeneveld 2024	134	15.8	29	137.6	20.4	28	8.1%	−3.60 [−13.10, 5.90]
Haack 2013	−14	3	13	−7	5	9	39.1%	−7.00 [−10.65, −3.35]
Johann 2020	0	13	23	−1	11	23	14.3%	1.00 [−5.96, 7.96]
Matthew 2023	109.18	10.87	11	116.18	12.45	11	7.7%	−7.00 [−16.77, 2.77]
**Subtotal (95% CI)**			**109**			**98**	**100.0%**	**−5.63 [−8.44, −2.82]**
Heterogeneity: $Tau^2 = 1.79$; $Chi^2 = 5.80$, df = 5 (P = 0.33); $I^2 = 14\%$								
Test for overall effect: Z = 3.92 (P < 0.0001)								
**Some concerns**								
Ham 2020	119.15	13.8	24	119.39	9.26	20	10.7%	−0.24 [−7.09, 6.61]
Hartescu 2021	−11.09	3.27	10	0.76	3.26	8	16.1%	−11.85 [−14.88, −8.82]
Javaheri 2020	−2.9	19.2	15	0.5	6.6	14	7.0%	−3.40 [−13.71, 6.91]
Li 2018	−5.3	3.2	16	−1.9	2.9	15	17.1%	−3.40 [−5.55, −1.25]
McGrath 2017	−0.9	9.4	54	−0.8	9.3	67	15.7%	−0.10 [−3.45, 3.25]
Reynold 2014	112.88	10.84	8	112.9	7.05	6	7.8%	−0.02 [−9.41, 9.37]
Stock 2020	111.2	14.1	53	117.1	19.1	53	11.3%	−5.90 [−12.29, 0.49]
Yang 2021	128.76	10.79	53	135.14	11.29	53	14.4%	−6.38 [−10.58, −2.18]
**Subtotal (95% CI)**			**233**			**236**	**100.0%**	**−4.35 [−7.81, −0.90]**
Heterogeneity: $Tau^2 = 16.93$; $Chi^2 = 33.33$, df = 7 (P < 0.0001); $I^2 = 79\%$								
Test for overall effect: Z = 2.47 (P = 0.01)								

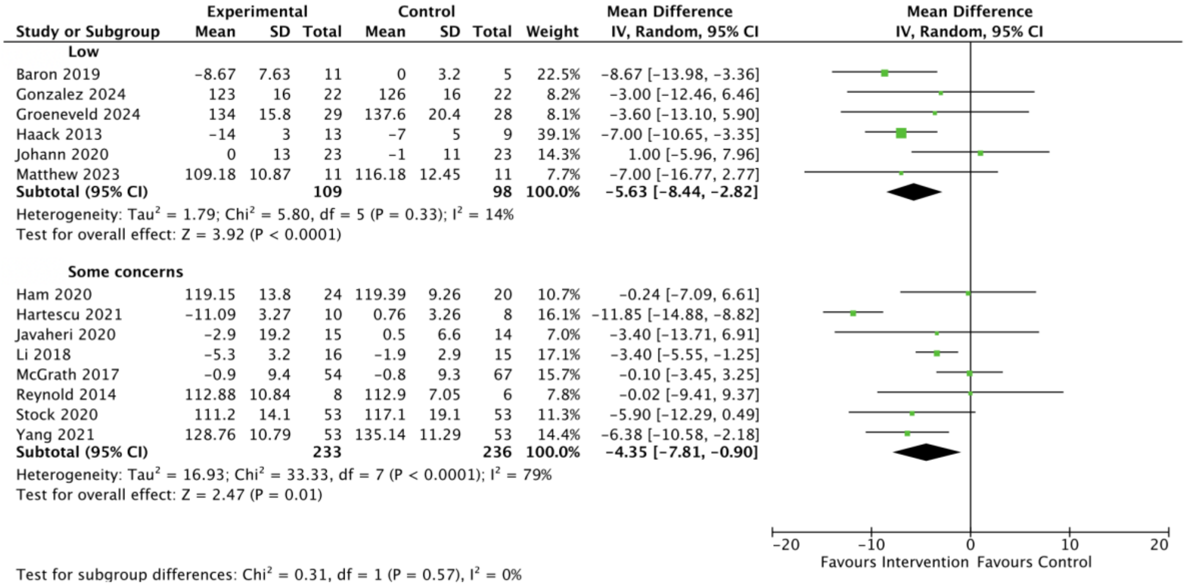

Test for subgroup differences: $Chi^2 = 0.31$, df = 1 (P = 0.57), $I^2 = 0\%$

(b) Diastolic blood pressure (mmHg)

	Experimental			Control				Mean Difference
Study or Subgroup	Mean	SD	Total	Mean	SD	Total	Weight	IV, Random, 95% CI
**Low**								
Baron 2019	−6	5.09	11	−1.83	2.51	5	19.0%	−4.17 [−7.90, −0.44]
Gonzalez 2024	72	7	22	73	7	22	16.3%	−1.00 [−5.14, 3.14]
Groeneveld 2024	75.8	8.4	29	76.9	6.4	28	18.0%	−1.10 [−4.97, 2.77]
Haack 2013	−8	3	13	−3	4	9	24.5%	−5.00 [−8.08, −1.92]
Johann 2020	0	9	23	−1	7	23	13.5%	1.00 [−3.66, 5.66]
Matthew 2023	62.09	6.27	11	66.36	8.05	11	8.8%	−4.27 [−10.30, 1.76]
**Subtotal (95% CI)**			**109**			**98**	**100.0%**	**−2.61 [−4.54, −0.69]**
Heterogeneity: $Tau^2 = 1.48$; $Chi^2 = 6.73$, df = 5 (P = 0.24); $I^2 = 26\%$								
Test for overall effect: Z = 2.66 (P = 0.008)								
**Some concerns**								
Ham 2020	69.72	7.84	24	69.31	7.2	20	12.3%	0.41 [−4.04, 4.86]
Hartescu 2021	−12.16	2.84	10	1.38	2.84	8	13.6%	−13.54 [−16.18, −10.90]
Javaheri 2020	−1.1	8.4	15	0.8	4.9	14	11.9%	−1.90 [−6.87, 3.07]
Li 2018	−2	4.1	16	−1.2	3.2	15	13.6%	−0.80 [−3.38, 1.78]
McGrath 2017	−0.6	6	54	−1.5	6.6	67	13.8%	0.90 [−1.35, 3.15]
Reynold 2014	71.42	8.62	8	73	8.42	6	8.6%	−1.58 [−10.58, 7.42]
Stock 2020	68.8	10.5	53	70.9	11.4	53	12.6%	−2.10 [−6.27, 2.07]
Yang 2021	78.86	7	53	78.3	7.52	53	13.5%	0.56 [−2.21, 3.33]
**Subtotal (95% CI)**			**233**			**236**	**100.0%**	**−2.33 [−6.47, 1.82]**
Heterogeneity: $Tau^2 = 31.04$; $Chi^2 = 84.07$, df = 7 (P < 0.00001); $I^2 = 92\%$								
Test for overall effect: Z = 1.10 (P = 0.27)								

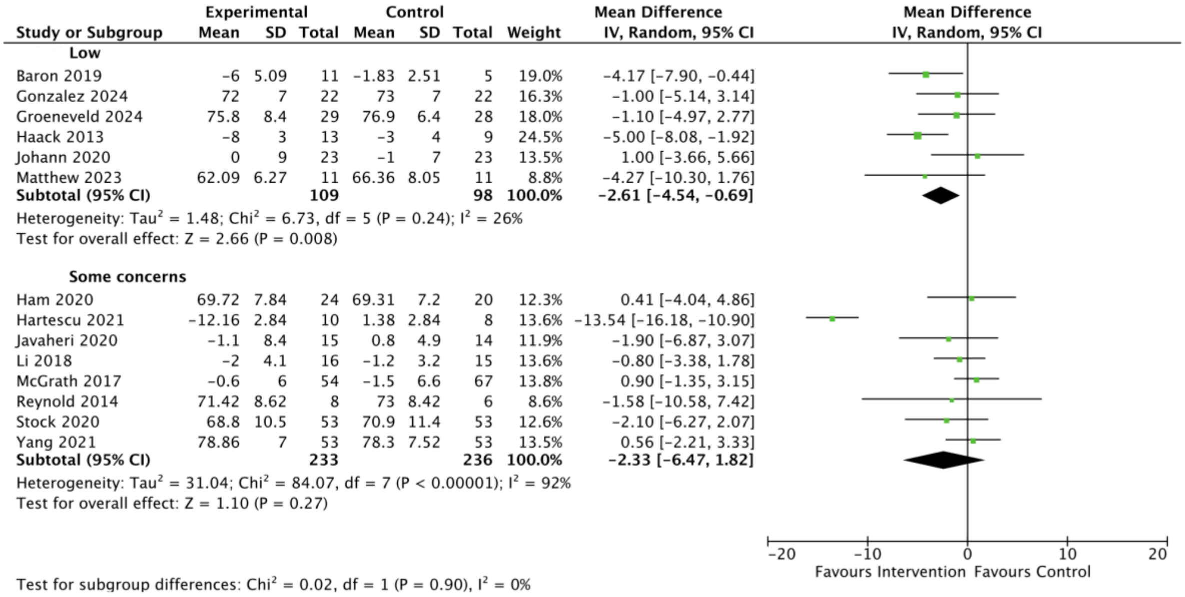

Test for subgroup differences: $Chi^2 = 0.02$, df = 1 (P = 0.90), $I^2 = 0\%$

Risk of bias categories were taken from Traffic-light plots in Appendix B. The category of ‘moderate concerns’ from ROBINS-I assessments was combined with ‘some concerns’ on ROB-2 assessments to allow for overall comparison in all studies. One study (Liu et al.) was not included in the ROB-2 analysis, hence did not appear in this sub-analysis. The test for sub-group differences was not significant for either systolic (p=0.57) or diastolic (p=0.90) blood pressure. Key: CI= confidence interval, I^2= heterogeneity test, IV = inverse variance, SD = standard deviation.

Appendix J. Results of the meta-analysis with a 'leave-one-out-analysis' for outcomes of a) systolic and b) diastolic blood pressure for all 15 studies

a) Systolic blood pressure (mmHg)

**Study omitted**	**N**	**Post-intervention change (95% CI), mmHg**	**P-value**	**I^2 (%)**
Total cohort (no omissions)	860	-5.02 (-7.23, -2.80)	0.0001	67
Baron 2019	844	-4.72 (-7.05, -2.39)	0.0001	68
Gonzalez 2024	816	-5.08 (-7.38, -2.79)	<0.0001	69
Groeneveld 2024	803	-5.06 (-7.36, 2.76)	<0.0001	69
Haack 2013	838	-4.78 (-7.21, -2.36)	<0.0001	68
Ham 2020	816	-5.30 (-7.58, -3.03)	0.0001	67
Hartescu 2021	842	-4.38 (-6.17, -2.59)	**0.06**	40
Javaheri 2020	831	-5.06 (-7.35, -2.77)	<0.0001	69
Johann 2020	814	-5.38 (-7.62, -3.13)	0.0002	66
Li 2018	829	-5.15 (-7.63, -2.68)	0.0003	65
Liu 2018	676	-4.70 (-7.12, -2.29)	0.0002	67
Matthew 2023	838	-4.93 (-7.22, -2.63)	<0.0001	69
McGrath 2017	739	-5.63 (-7.78, -3.49)	0.003	59
Reynold 2014	846	-5.21 (-7.48, -2.94)	0.0001	68
Stock 2020	754	-4.94 (-7.28, -2.59)	<0.0001	69
Yang 2021	754	-4.86 (-7.27, -2.45)	<0.0001	69

b) Diastolic blood pressure (mmHg)

**Study omitted**	**N**	**Post-intervention change (95% CI), mmHg**	**P-value**	**I^2 (%)**
Total cohort (no omissions)	860	-2.90 (-5.49, -0.30)	0.03	88
Baron 2019	844	-2.79 (-5.57, -0.02)	0.05	89
Gonzalez 2024	816	-3.03 (-5.76, -0.29)	0.03	89
Groeneveld 2024	803	-3.02 (-5.77, -0.27)	0.03	89
Haack 2013	838	-2.72 (-5.52, 0.06)	**0.06**	89
Ham 2020	816	-3.12 (-5.38, -0.41)	0.02	88
Hartescu 2021	842	-2.07 (-4.02, -0.13)	0.04	75
Javaheri 2020	831	-2.96 (-5.68, -0.23)	0.03	89
Johann 2020	814	-3.16 (-5.85, -0.46)	0.02	88
Li 2018	829	-3.05 (-5.86, -0.25)	0.03	88
Liu 2018	676	-2.39 (-4.96, 0.17)	**0.07**	86
Matthew 2023	838	-2.81 (-5.52, -0.10)	0.04	89
McGrath 2017	739	-3.21 (-5.90, -0.52)	0.02	87
Reynold 2014	846	-2.95 (-5.62, -0.28)	0.03	89
Stock 2020	754	-2.95 (-5.70, -0.20)	0.04	89
Yang 2021	754	-31.7 (-5.90, -0.44)	0.02	88

Key: CI = confidence intervals, I^2 = I^2 for heterogeneity, mmHg = millimetres of mercury, N = number of participants. In both cases, the exclusion of one study at a time revealed consistent and significant reductions for both systolic and diastolic blood pressure ($p<0.05$), with the exceptions of (a) Hartescu et al. for systolic blood pressure and (b) Liu et al. and Haack et al., for diastolic blood pressure.

Appendix K. Sub-analysis of the main meta-analysis outcomes for systolic and diastolic blood pressure according to baseline sleep-problem phenotype.

a) Table of classifications of studies within the meta-analysis (n=15) according to the baseline sleep-problem phenotype.

Sleep-problem phenotype	Criteria	Studies
**Insomnia**	Insomnia severity index score > 10 or fulfilling criteria for International Classification of Sleep Disorders.	Groeneveld, Ham, Johann, Javaheri, McGrath, Yang
**Short sleep**	Sleep duration < 6.5 hours (or time-in-bed/ total sleep time <7 hours) $	Baron, Gonzalez, Haack, Hartescu, Li, Mathew, Reynold, Stock,
**Clinically significant sleep disorder**	A sleep problem detected (e.g. PSQI score >5) without further qualification of exact problem.	Liu

$ definitions made for the purposes of this study (other definitions exist, but those employed here were the most appropriate definitions given the data available). Key: PSQI = Pittsburgh Sleep Quality Index. Each study was allocated to one category only based on the available data in individual studies; however, in theory as sleep problems tend to overlap, it is possible some studies could have fitted into more than one category if all sleep variables were measured. Sleep extension studies focused more on measuring sleep duration/ time-in-bed and less on PSQI global and insomnia scores and vice-versa for CBT-I and/ or sleep hygiene studies.

b) Forrest plots for the results of the meta-analysis examining the effect of sleep interventions according to baseline sleep-problem phenotype comparing short sleep and insomnia for outcomes of (i) systolic and (ii) diastolic blood pressure

i) Systolic blood pressure (mmHg)

Study or Subgroup	Experimental Mean	Experimental SD	Experimental Total	Control Mean	Control SD	Control Total	Weight	Mean Difference IV, Random, 95% CI
**Insomnia**								
Groeneveld 2024	134	15.8	29	137.6	20.4	28	7.4%	−3.60 [−13.10, 5.90]
Ham 2020	119.15	13.8	24	119.39	9.26	20	12.9%	−0.24 [−7.09, 6.61]
Javaheri 2020	−2.9	19.2	15	0.5	6.6	14	6.4%	−3.40 [−13.71, 6.91]
Johann 2020	0	13	23	−1	11	23	12.6%	1.00 [−5.96, 7.96]
McGrath 2017	−0.9	9.4	54	−0.8	9.3	67	34.3%	−0.10 [−3.45, 3.25]
Yang 2021	128.76	10.79	53	135.14	11.29	53	26.4%	−6.38 [−10.58, −2.18]
**Subtotal (95% CI)**			**198**			**205**	**100.0%**	**−2.11 [−4.83, 0.62]**
Heterogeneity: Tau² = 2.70; Chi² = 6.55, df = 5 (P = 0.26); I² = 24%								
Test for overall effect: Z = 1.52 (P = 0.13)								
**Short sleep**								
Baron 2019	−8.67	7.63	11	0	3.2	5	13.2%	−8.67 [−13.98, −3.36]
Gonzalez 2024	123	16	22	126	16	22	7.2%	−3.00 [−12.46, 6.46]
Haack 2013	−14	3	13	−7	5	9	16.6%	−7.00 [−10.65, −3.35]
Hartescu 2021	−11.09	3.27	10	0.76	3.26	8	17.9%	−11.85 [−14.88, −8.82]
Li 2018	−5.3	3.2	16	−1.9	2.9	15	19.6%	−3.40 [−5.55, −1.25]
Matthew 2023	109.18	10.87	11	116.18	12.45	11	6.9%	−7.00 [−16.77, 2.77]
Reynold 2014	112.88	10.84	8	112.9	7.05	6	7.3%	−0.02 [−9.41, 9.37]
Stock 2020	111.2	14.1	53	117.1	19.1	53	11.3%	−5.90 [−12.29, 0.49]
**Subtotal (95% CI)**			**144**			**129**	**100.0%**	**−6.47 [−9.57, −3.36]**
Heterogeneity: Tau² = 11.61; Chi² = 22.98, df = 7 (P = 0.002); I² = 70%								
Test for overall effect: Z = 4.08 (P < 0.0001)								

Test for subgroup differences: Chi² = 4.28, df = 1 (P = 0.04), I² = 76.6%

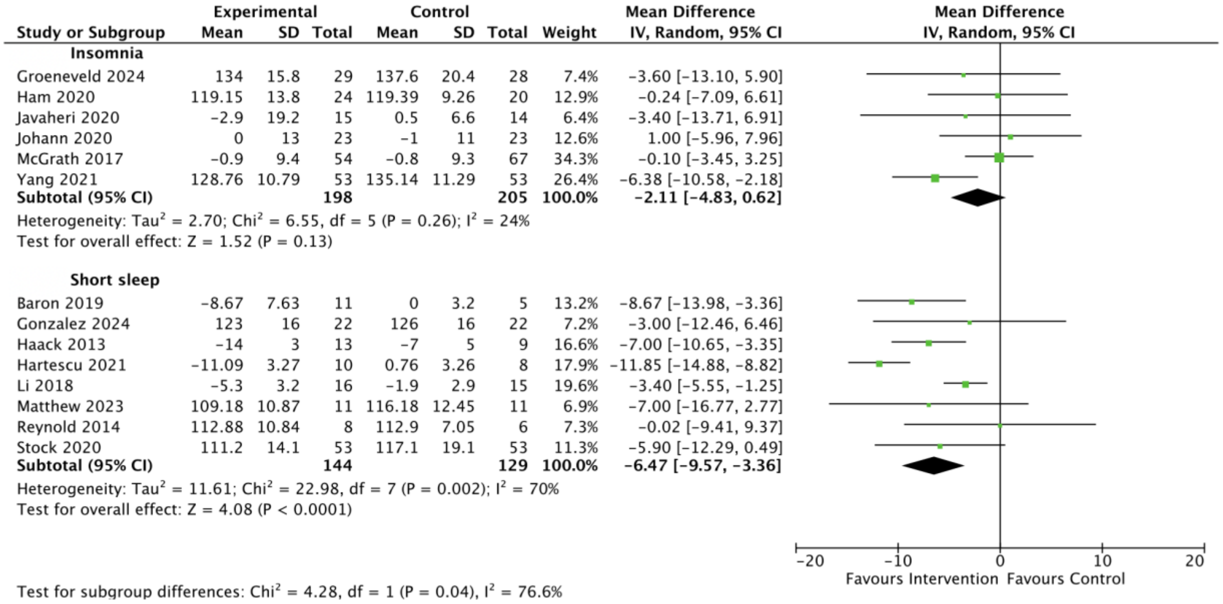

ii) Diastolic blood pressure (mmHg)

Study or Subgroup	Experimental Mean	Experimental SD	Experimental Total	Control Mean	Control SD	Control Total	Weight	Mean Difference IV, Random, 95% CI
**Insomnia**								
Groeneveld 2024	75.8	8.4	29	76.9	6.4	28	12.6%	−1.10 [−4.97, 2.77]
Ham 2020	69.72	7.84	24	69.31	7.2	20	9.5%	0.41 [−4.04, 4.86]
Javaheri 2020	−1.1	8.4	15	0.8	4.9	14	7.6%	−1.90 [−6.87, 3.07]
Johann 2020	0	9	23	−1	7	23	8.7%	1.00 [−3.66, 5.66]
McGrath 2017	−0.6	6	54	−1.5	6.6	67	37.1%	0.90 [−1.35, 3.15]
Yang 2021	78.86	7	53	78.3	7.52	53	24.6%	0.56 [−2.21, 3.33]
**Subtotal (95% CI)**			**198**			**205**	**100.0%**	**0.31 [−1.06, 1.68]**
Heterogeneity: $Tau^2 = 0.00$; $Chi^2 = 1.65$, df = 5 (P = 0.89); $I^2 = 0\%$								
Test for overall effect: Z = 0.45 (P = 0.65)								
**short sleep**								
Baron 2019	−6	5.09	11	−1.83	2.51	5	13.2%	−4.17 [−7.90, −0.44]
Gonzalez 2024	72	7	22	73	7	22	12.8%	−1.00 [−5.14, 3.14]
Haack 2013	−8	3	13	−3	4	9	13.8%	−5.00 [−8.08, −1.92]
Hartescu 2021	−12.16	2.84	10	1.38	2.84	8	14.1%	−13.54 [−16.18, −10.90]
Li 2018	−2	4.1	16	−1.2	3.2	15	14.1%	−0.80 [−3.38, 1.78]
Matthew 2023	62.09	6.27	11	66.36	8.05	11	11.0%	−4.27 [−10.30, 1.76]
Reynold 2014	71.42	8.62	8	73	8.42	6	8.2%	−1.58 [−10.58, 7.42]
Stock 2020	68.8	10.5	53	70.9	11.4	53	12.8%	−2.10 [−6.27, 2.07]
**Subtotal (95% CI)**			**144**			**129**	**100.0%**	**−4.26 [−8.06, −0.46]**
Heterogeneity: $Tau^2 = 24.83$; $Chi^2 = 56.58$, df = 7 (P < 0.00001); $I^2 = 88\%$								
Test for overall effect: Z = 2.20 (P = 0.03)								

Test for subgroup differences: $Chi^2 = 4.92$, df = 1 (P = 0.03), $I^2 = 79.7\%$

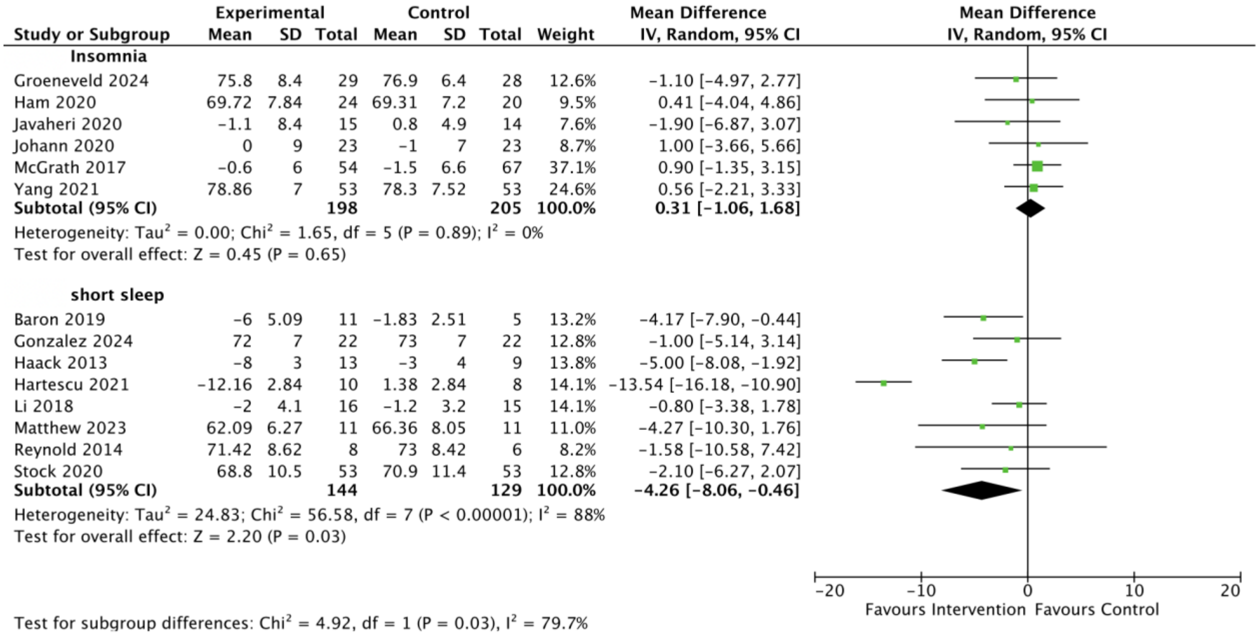

Key: CI= confidence interval, I^2= heterogeneity test, IV = inverse variance, SD = standard deviation. Sleep-problem phenotypes containing two or more studies were included in the Forrest plots.

Appendix L. GRADE assessment summary of findings

Assessment	Risk of Bias	Inconsistency of results	Indirectness	Imprecision	Publication bias	Final certainty
Systolic blood pressure	Some concerns	Inconsistent	Low risk	Some degree	Low risk	Low
Diastolic blood pressure	Some concerns	Inconsistent	Low risk	Some degree	Low risk	Low

Key: GRADE = Grading of recommendations, assessments, development and evaluation.
